# Regulatory Mechanism of M1/M2 Macrophage Polarization in the Development of Autoimmune Diseases

**DOI:** 10.1155/2023/8821610

**Published:** 2023-06-08

**Authors:** Yuan Peng, Mengxian Zhou, Hong Yang, Ruyi Qu, Yan Qiu, Jiawen Hao, Hongsheng Bi, Dadong Guo

**Affiliations:** ^1^Shandong University of Traditional Chinese Medicine, Jinan 250002, China; ^2^Qingdao Traditional Chinese Medicine Hospital (Qingdao Hiser Hospital), Qingdao 266033, China; ^3^The Second Affiliated Hospital of Shandong University of Traditional Chinese Medicine, Jinan 250002, China; ^4^Shandong Provincial Key Laboratory of Integrated Traditional Chinese and Western Medicine for Prevention and Therapy of Ocular Diseases; Shandong Academy of Eye Disease Prevention and Therapy, Medical College of Optometry and Ophthalmology, Shandong University of Traditional Chinese Medicine, Jinan 250002, China

## Abstract

Macrophages are innate immune cells in the organism and can be found in almost tissues and organs. They are highly plastic and heterogeneous cells and can participate in the immune response, thereby playing a crucial role in maintaining the immune homeostasis of the body. It is well known that undifferentiated macrophages can polarize into classically activated macrophages (M1 macrophages) and alternatively activated macrophages (M2 macrophages) under different microenvironmental conditions. The directions of macrophage polarization can be regulated by a series of factors, including interferon, lipopolysaccharide, interleukin, and noncoding RNAs. To elucidate the role of macrophages in various autoimmune diseases, we searched the literature on macrophages with the PubMed database. Search terms are as follows: macrophages, polarization, signaling pathways, noncoding RNA, inflammation, autoimmune diseases, systemic lupus erythematosus, rheumatoid arthritis, lupus nephritis, Sjogren's syndrome, Guillain-Barré syndrome, and multiple sclerosis. In the present study, we summarize the role of macrophage polarization in common autoimmune diseases. In addition, we also summarize the features and recent advances with a particular focus on the immunotherapeutic potential of macrophage polarization in autoimmune diseases and the potentially effective therapeutic targets.

## 1. Introduction

Autoimmune diseases are chronic, refractory clinical common diseases that are mainly stimulated by immune, hormonal, environmental, and genetic factors [1]. To date, there are approximately 150 autoimmune diseases, and the prevalence is increasing year by year. Clinically, most autoimmune diseases are chronic that exist for a long time or even accompany patients for life, and there is still no specific treatment method [2]. Autoimmune diseases seriously affect patients' health, so it is urgent to find effective treatment strategies to improve their quality of life. However, the pathogenesis of at least half of autoimmune diseases is unclear. The immune system is believed to lose tolerance to autoantigens, produce excess autoantibodies against antigens, overrespond to immune cells, attack its tissues and organs, and cause cell damage or abnormal function, resulting in local or systemic inflammation and tissue damage [3]. Autoantigens may include viruses, abnormally deposited immune complexes, extra neutrophil traps, or excess apoptotic substances [4]. Macrophages are an essential part of the innate immune system, which exists in almost all body tissues, contribute to immune regulation and tissue repair, and maintain immune homeostasis [5]. Macrophages are the dominant immune cell population at all disease stages, and their dysfunction can lead to abnormal repair and regeneration, with runaway production of inflammatory mediators and growth factors [6].

Clear and convincing evidence has shown that macrophages are highly plastic and can polarize into different types of macrophages under different microenvironment conditions: classically activated macrophages (M1) and alternatively activated macrophages (M2), a traditional classification, and a simplified, old classification method. Among them, we prefer to interpret M2-type macrophages as activated macrophages other than M1. M1 can be stimulated and activated by lipopolysaccharide (LPS) and interferon- (IFN-) *γ*; can secrete inflammatory factors such as tumor necrosis factor-*α* (TNF-*α*), interleukin(IL)-1*β*, and IL-6; and can generate a large number of reactive oxygen species (ROS) and reactive nitrogen species (RNS), killing invading pathogens, phagocytose, and clear senescent, damaged, and degenerated cells [7, 8]. M2 can be activated by IL-4 and IL-13; can secrete anti-inflammatory cytokines including IL-10, IL-4, and transforming growth factor-*β* (TGF-*β*); can also produce vascular endothelial growth factor (VEGF) and platelet-derived growth factor (PDGF); can inhibit T cell proliferation and activation; and can participate in T helper (Th) 2-type immunity response, contributing to tissue repair and angiogenesis [9, 10]. However, the excessive accumulation of M2 macrophages is closely related to fibrosis [11]. Fibrosis results from excessive accumulation of extracellular matrix (ECM) components such as collagen and fibronectin in dysregulated tissue repair after injury [12]. ECM can promote wound healing and tissue repair when minor tissue damage occurs. By contrast, if the injury is severe, excessive accumulation of ECM can destroy tissue structure and lead to organ dysfunction [12]. Thus, macrophages play an essential role in the development of fibrosis [13], of which M2a macrophages can significantly promote fibrosis progression [14].

Macrophage polarization is a dynamic and reversible process involved in the occurrence and development of many autoimmune diseases, such as uveitis, systemic lupus erythematosus, rheumatoid arthritis, and Sjogren's syndrome [15–19]. Studies have revealed that regulating the balance of M1/M2 macrophage polarization can control the inflammatory progression of autoimmune diseases, exhibiting an excellent therapeutic effect on alleviating inflammatory damage and helping extracellular matrix remodeling in autoimmune diseases. This review mainly describes the role of macrophage polarization and the research progress of polarized macrophages as therapeutic targets in autoimmune diseases.

## 2. Materials and Methodology

A search strategy was performed to extract the available literature using the PubMed database. The search terms “macrophages,” “polarization,” “signaling pathways,” “noncoding RNA,” and “inflammation” combined with terms like autoimmune diseases such as systemic lupus erythematosus, rheumatoid arthritis, lupus nephritis, Sjogren's syndrome, Guillain-Barre syndrome, and multiple sclerosis were searched. Original researches, including prospective and retrospective studies and review papers, were included and cross-referenced.

## 3. Results and Discussion

### 3.1. Origin and Function of Macrophages

In 1908, Nobel Prize winner Ilya Metchnikov discovered cells with phagocytosis from single-celled organisms to vertebrates and called them macrophages [20]. Throughout life, the primary role of macrophages is to phagocytose cell debris and recycle red blood cells damaged by aging, which is also linked to iron metabolism [21]. Initially, there was a certain misunderstanding about the origin of macrophages, and it was believed that macrophages could be continuously replenished entirely by monocytes. Subsequently, researchers believe that there are two primary sources of macrophages: the first source is that monocytes originating from bone marrow hematopoietic stem cells enter the tissue to form macrophages; the second source is the progenitor cells of the embryonic yolk sac, which develop into tissue-resident macrophages with local self-renewal capacity and exist independently of monocytes in adulthood [22–27]. As a type of phagic cell, macrophages in the immune system can engulf and kill foreign bacteria, pathogens, and cells damaged by self-aging, participating in the body's second line of defense. At the same time, macrophages can participate in antigen processing and presentation, activating humoral immunity and cellular immunity to initiate an adaptive immune response [28]. As a result, macrophages can resist pathogen attacks and play an essential role in the transmission of immune information, tissue development, and in maintaining the body's homeostasis.

### 3.2. Tissue Distribution of Macrophages

According to the distribution of different physiological locations, macrophages are mainly found in lymph nodes, alveolar walls, bone marrow, liver, spleen, and other organs. They can be divided into subgroups, including microglia in the central nervous system, osteoclasts, alveolar macrophages in the lung, histiocytes in the spleen and interstitial connective tissue, and Kupffer cells in the liver [26]. Macrophages in different parts are closely related to diseases in corresponding organs. For example, Kupffer cells in the hepatic sinuses can renew themselves and play a central role in acute and chronic liver failure, liver fibrosis, nonalcoholic fatty liver disease, alcoholic liver disease, viral hepatitis, and hepatocellular carcinoma pathogenesis, as well as disease remission [10]. The central nervous system (CNS) microglia can influence neuronal postinjury properties by regulating the clearance of myelin and cell debris and cytokine release [29]. Zabala et al. reported that blocking microglial P2X4 signaling can exacerbate the clinical symptoms of experimental autoimmune meningitis models, thereby contributing to microglial activation into a proinflammatory phenotype [30].

### 3.3. Metabolic Characteristics of Macrophages

Shifts in cellular metabolism are closely related to phenotypic and functional changes in macrophages. M1-type macrophage metabolism mainly depends on glycolysis, followed by the pentose phosphate pathway (PPP), truncated tricarboxylic acid cycle (TCA cycle), and dysfunctional mitochondrial oxidative phosphorylation (OXPHOS) [31]. In contrast, M2 cells depend more on OXPHOS and the TCA cycle because their TCA cycle is intact and can provide substrates for the electron transport chain (ETC) complex [32]. Recent advances in research have focused mainly on amino acids and related metabolic pathways that promote macrophage polarization, such as iNOS/ARG1 (arginine 1), the TCA cycle and OXPHOS (glutamine), and single-carbon metabolism (serine and glycine) [33]. Mitochondrial metabolism is critically linked to macrophage polarization. Mitochondria provide energy for cells and coordinate signal transduction, chromatin regulation, and transcriptional regulation to influence macrophage polarization by fine-tuning the dynamic signals of the immune response [34]. So, we speculate that those factors influencing macrophage metabolism may disrupt M1/M2 homeostasis and exacerbate inflammation. It has been shown that mitochondrial functional impairment can promote reactive oxygen species (ROS) production, increase hypoxia-inducible factor 1-*α* (HIF1-*α*) expression, and decrease mitochondrial complexes I and IV of OXPHOS, which in turn affect the reprogramming of macrophages in glucose metabolism [35]. Therefore, it is meaningful to focus on the role of glucose metabolism reprogramming of M1 macrophages in inflammatory initiation and to inhibit inflammation by blocking glucose metabolism reprogramming. Researchers have identified a novel role in macrophage activation-related inflammation, where metabolic reprogramming occurs after macrophage exposure to inflammatory stimuli [36]. Itaconate, a mitochondrial metabolite in metabolic reprogramming, can inhibit succinate dehydrogenase and multiple levels of glycolysis, activate anti-inflammatory transcription factors nuclear factor E2-related factor 2 (Nrf2) and activating transcription factor 3 (ATF3), and inhibit NLR family pyrin domain containing 3 (NLRP3) inflammasome [36–38]. Accordingly, the production of proinflammatory mediators of M1 macrophages under LPS stimulation can be significantly reduced, damage can be ameliorated, and tissue repair can be promoted [36].

### 3.4. Macrophage Polarization

Mature macrophages undergo phenotypic and morphological differentiation under various factors (e.g., pathogen invasion, tissue damage, and metabolic disturbances) and termed macrophage polarization. In the early stage, macrophages are mainly divided into classically/inflammatory activated macrophages (M1 macrophages) and alternatively/wound healing-activated macrophages (M2 macrophages), which are the two terminal states of macrophage polarization depending on their polarization status and function. Later, some scholars referred to the basic principle of the three primary colors in the color wheel and divided macrophages into classically activated macrophages, wound healing macrophages, regulated macrophages, and hybrid macrophage groups with the characteristics of the above two macrophages at the same time [39]. But in the new nomenclature proposed by Murray et al., it is also proposed to avoid the term “regulatory macrophage” and recommend describing the stimulation scene and stimulator to define the macrophage activation state [40]. Therefore, although the “M1-M2 paradigm” is a more extreme classification method, it is still being used for the convenience of distinction, which is not a strict functional classification, but a simplified operating concept [25, 41]. The detailed subtypes and functions of M2 macrophages are also distinguished below. It is clear that LPS, IFN-*γ*, TNF-*α*, and granulocyte-macrophage colony-stimulating factor (GM-CSF) stimulation activate M1 macrophages (M1 biomarkers: CD86, CD40, and CD38) [42], and M1 macrophages secrete proinflammatory factors such as IL-12, IL-6, IL-1*β*, IL-23, IFN-*γ*, and TNF-*α*, producing a large number of ROS and inducible nitric oxide synthase (iNOS) [43–45]. Therefore, they can promote the inflammatory response, resist pathogens, and inhibit the occurrence and development of tumors [46, 47]. Prostaglandins E2 (PGE2), IL-13, IL-4, and macrophage colony-stimulating factor (M-CSF) can activate M2 macrophages (M2 biomarkers: CCL7, CCL17, CCL22, CCL24, CD83, and CD44 [48, 49]. In addition, M2 macrophages can also inhibit the inflammatory response and promote tissue remodeling and tumor progression by producing anti-inflammatory factor IL-10, transforming growth factor-*β* (TGF-*β*), peroxisome proliferator-activated receptor *γ* (PPAR*γ*), arginase 1 (Arg1), Fizz1, and Ym1 [9, 50–52]. M2 macrophages produce transcriptional changes under different stimulation conditions, which can be subdivided into M2a, M2b, M2c, and M2d (Table 1).

M2a induced by IL-4 and IL-13 is called wound healing macrophage, which highly expresses CD206, IL-1R, and CCL17 and secretes fibronectin and other fibrogenic molecules, promoting tissue repair and remodeling [45, 53–56]. Immune complexes, toll-like receptor (TLR) agonists, and IL-1R stimulate the production of M2b (regulating macrophages) that highly express CCL1 and TNFSF14. M2b can secrete anti-inflammatory and proinflammatory factors, mainly involved in immune regulation and Th2 activation [45, 54–58]. Glucocorticoids, TGF-*β*, and IL-10 can induce the production of M2c, which is characterized by CD163 on the surface and secretes IL-10 and TGF-*β*1 and participates in inhibiting the immune response and tissue remodeling [45, 55, 56, 58, 59]. TLR, adenosine receptor, and IL-6-induced M2d (tumor-associated macrophages) play a significant role in angiogenesis and tumor-related progression [45, 55, 56, 60–64]. However, studies have also found that in mouse models, although there is some overlap between in vivo M1 (LPS+) and in vitro classical activation and in vivo M2 (LPS-) and in vitro alternatively activated macrophages, more genes are regulated in opposite or unrelated ways. For example, chemokine CCL2, hematopoietic cytokine Csf2 (GM-CSF), IL-15, IL-23a, and IFN*β*1 positively correlate with M1 polarization in vivo but do not increase in vitro classically activated macrophages [65]. Those can explain that in vitro classical and alternative macrophage activation does not match M1/M2 polarization in vivo, suggesting that we should pay attention to the nonnegligible differences in macrophages in different environments (in vivo/in vitro).

### 3.5. Macrophage Polarization and Noncoding RNAs

In addition to IFN-*γ*, LPS, IL-4, TNF-*α*, GM-CSF-1, and M-CSF, noncoding RNAs can also regulate macrophage polarization. Noncoding RNA refers to RNA that participates in the transcription process of genes but does not translate proteins, mainly including microRNA (miRNA), long noncoding RNA (lncRNA), and circular RNA (circRNA) [66]. Noncoding RNA can affect the polarization balance of macrophages through different mechanisms of action.

miRNAs are evolutionarily highly conserved noncoding RNAs with a length of about 22 nucleotides involved in immune responses and tumor growth regulation. Currently, a large number of miRNAs have been identified, of which some can regulate macrophage polarization, such as miR-156a, miR-33, miR-let-7a, miR-223, miR-155, miR-21, miR-125a, miR-19b-3p, miR-100-5p, miR-654, and miR-221-3p [67–79], as shown in Table 2.

lncRNAs are non-protein-coding RNAs with a length of more than 200 nucleotides, which are involved in cell differentiation and proliferation, cycle regulation, tumor development, and other pathophysiological processes. Macrophage polarization is also affected by some lncRNAs, such as ANCR, Mirt2, HITT, GAS5, and RN7S [80–86], as shown in Table 3.

In addition, circRNAs are a new hotspot in the field of RNA research and are a special noncoding RNA. They have also been confirmed in the latest study to promote macrophage polarization to M2 and thus participate in the regulation of disease. circSAFB2 mediates M2 macrophage polarization through the miR-620/JAK1/STAT3 axis [87, 88], while circACTR2 activates YAP signaling by targeting miR-200c, stimulates M2 macrophage polarization, and promotes renal fibrosis [89]. Nevertheless, it is still unclear in the mechanism of circular RNA-mediated macrophage polarization and needs to be further explored. Currently, it has reached some consensus that noncoding RNAs play an essential role in macrophage polarization, and these noncoding RNAs can serve as effective biomarkers for disease diagnosis and therapeutic targets.

#### 3.5.1. Noncoding RNAs and Systemic Lupus Erythematosus

An in vitro study confirmed that lncRNA-GAS5 expression is downregulated in SLE [90]; meanwhile, knockdown of lncRNA-RN7SK and lncRNA-GAS5 can downregulate the levels of M2 markers (CD163, CD206, or Dectin) and upregulate the levels of M1 markers (MHC II or CD23), confirming that both lncRNA-RN7SK and lncRNA-GAS5 can promote M2 macrophage polarization and therefore exert a mitigating effect on SLE [80, 90]. Nevertheless, how noncoding RNA regulates macrophage polarization to affect the pathogenesis of SLE is still unclear.

#### 3.5.2. Noncoding RNAs and Lupus Nephritis

It is found that lncRNA NEAT1 is up-regulated in SLE patients and can enhance the expression of cytokines or chemokines such as IL-6, CCL2, and CXCL10 by phosphorylating JNK and ERK. These cytokines can attract Th1 cells and participate in the pathogenesis of LN [91]. Meanwhile, it is also confirmed that lncRNA NEAT1 participates in TLR-mediated inflammatory response in monocytes through the MAPK pathway [92]. MicroRNA-382 can activate the STAT3 signaling pathway by downregulating signal regulatory protein *α* (SIRP-*α*) to promote M2-type macrophages, and sustained M2 macrophage infiltration promotes renal fibrosis. However, microRNA-382 knockout or M2 macrophage depletion can inhibit the expression of *α*-SMA, fibronectin, and collagen I and exhibit a certain mitigating effect on kidney fibers [76], indicating that miR-382 may become a promising therapeutic target in the future.

#### 3.5.3. Noncoding RNAs and Rheumatoid Arthritis

In the synovial chamber of RA patients, the expression of miR-221-3p is abnormally increased, and M2 macrophages are transferred to proinflammatory M1 through the JAK3/STAT3 pathway, promoting the development of joint inflammation [77]. In an in vitro cell experiment in RA patients, the drug tocilizumab has a certain alleviating effect on inflammation, mainly by inducing lncRNA MIR31HG, reducing miR-214, inhibiting the macrophage AKT pathway, and decreasing proinflammatory M1 macrophage frequency, thereby exhibiting a certain protective effect on chondrocytes [93].

#### 3.5.4. Noncoding RNAs and Multiple Sclerosis

lncRNA GAS5 inhibits M2 polarization and intensifies demyelinating by inhibiting transcription of TRF2, a critical factor in M2 macrophage polarization, and interfering with microglial GAS5 in vitro experiments can attenuate the progression of experimental autoimmune encephalomyelitis (EAE) [85]. At the same time, the latest research found that miR-467f and miR-466q can inhibit the expression of Map3k8 and MK2 and attenuate the proinflammatory phenotype of microglia through the p38 MAPK signaling pathway, thus having a good alleviating effect on the neuroinflammation of MS [78].

#### 3.5.5. Noncoding RNAs and Autoimmune Uveitis

In addition, microRNAs are closely associated with autoimmune uveitis. miRNAs can participate in the inflammatory or inflammatory mitigation process of uveitis by acting on signaling molecules of the NF-*κ*B pathway in macrophage polarization [94]. The toll-like receptor (TLR4) in the NF-*κ*B pathway can recruit MyD88 and IRAK after LPS stimulation, and IRAK phosphorylation activates NF-*κ*B after interacting with TRAF6 to promote M1 production. In an animal model of endotoxin-induced uveitis (EIU), miR-93 was found to bind to IRAK4 in the NF-*κ*B pathway, thereby inhibiting NF-*κ*B activation and thus negatively regulating the generation of M1-type macrophage-related proinflammatory cytokines [79]. miR-30b-5p is downregulated in the spleen, lymph nodes, and eye tissues of rats with autoimmune uveitis, and both in vitro and in vitro experiments have confirmed that supplementation with miR-30b-5p can reduce the level of IL-10 and TLR4-positive cells, thereby having a certain inhibitory effect on uveitis [95]. Meanwhile, miR-155 has been shown to promote M1 macrophage polarization and thus exert proinflammatory effects, showing remission to EAU after reducing miR-155 expression levels [67, 96]. However, how miR-155 regulates the polarization of macrophages by regulating the expression of target genes and then affects the pathogenesis of uveitis needs to be further investigated.

#### 3.5.6. Noncoding RNAs and Sjogren's Syndrome

Interestingly, studies have shown that different lncRNAs and miRNAs are differentially expressed in primary SS and participate in the pathogenic process of the disease [97, 98]. A rabbit model of autoimmune dacryoadenitis found that small extracellular vesicles derived from human umbilical cord mesenchymal stem cells promote M2 macrophage polarization and induce Tregs by miR-100-5p, thereby alleviating autoimmune dacryoadenitis [99].

Collectively, a deep understanding of the mechanism of noncoding RNAs regulating macrophage polarization under different conditions can more effectively help us manipulate the expression and silence these noncoding RNAs through drug targeting to control the direction of macrophage polarization, opening up a new horizon for the treatment of inflammatory diseases.

### 3.6. Macrophage Polarization and Autoimmune Diseases

#### 3.6.1. Profiling of Autoimmune Disease

Autoimmune diseases are clinical diseases caused by the destruction of autoimmune tolerance or abnormal regulation of autoimmune cells, the continuous immune response of the immune system to autoantigens, and the damage or dysfunction of self-organizing cells induced by some genetic and environmental factors [100].

Notably, age and gender are the related factors that induce autoimmune diseases. Most autoimmune diseases can occur at any age; however, some autoimmune disorders mainly occur in childhood and adolescence (such as type I diabetes), middle adulthood (such as myasthenia gravis and multiple sclerosis), or the elderly (such as rheumatoid arthritis and primary systemic vasculitis) [101]. The incidence rate of autoimmune diseases accounts for approximately 5-10% of the total population. For most autoimmune diseases, there are significant gender differences in prevalence, and women are usually affected more frequently than men. For example, women are about nine times more likely to develop systemic lupus erythematosus (SLE) than men, dramatically impacting the quality of life of female patients [102, 103]. This sex difference may contribute to estrogen. After estrogen binds to immune cell receptors, it can participate in the regulation of transcription factors, such as activating protein 1 (AP-1) and NF-*κ*B as a cofactor, possessing a certain pathogenic effect on SLE and multiple sclerosis (MS) [104]. Currently, the main goal of treating autoimmune diseases is to alleviate inflammation, relieve symptoms, attenuate organ damage, and minimize the possibility of recurrence [105], prolonging patients' survival time and optimizing the individual quality of life.

#### 3.6.2. Macrophage Polarization and Systemic Lupus Erythematosus

SLE is an autoimmune connective tissue disease that often involves multiple systems, with a wide range of clinical manifestations. It usually occurs in young women aged between 20 and 40. At present, the potential incidence rate and mortality are significant, and the pathogenesis and etiology are still unclear [106, 107]. Clinically, the diagnosis of SLE is mainly based on the combination of typical clinical manifestations and serological positivity, and characteristic clinical manifestations include cutaneous lupus erythematosus, alopecia, joint pain caused by musculoskeletal involvement, proteinuria caused by kidney involvement, and mental abnormalities caused by central nervous system involvement (seizures, psychosis, and coma) [108]. Currently, there are four main types of treatment for SLE: nonsteroidal anti-inflammatory drugs, antimalarial drugs, glucocorticoids, and immunosuppressive drugs for heavier forms of the disease [109]. Organ-threatening or life-threatening SLE usually includes initial high-intensity immunosuppressive therapy to control disease activity, followed by long-term low-intensity therapy to consolidate the response and prevent recurrence [110].

The pathogenesis of SLE is not only related to the abnormality of B cells and T cells but also related to inflammation-promoting M1 and immunosuppressive M2. It is confirmed that biological factors that promote the polarization of M1 macrophages will exacerbate the inflammation of SLE, and M2 is also involved in the pathogenesis of SLE. Human cytomegalovirus (HCMV) has been shown to be one of the major factors that can trigger SLE. In a clinical study involving SLE patients, HCMV infection-associated human cytomegalovirus protein (US31) was elevated in SLE patients and promoted NF-*κ*B2 activation, leading to M1 macrophage polarization and further deterioration of SLE [111]. In addition, a recent in vitro experiment found that toll-like receptor 7 and toll-like receptor 9 (TLR7 and TLR9) agonists can activate peritoneal macrophages to secrete higher levels of proinflammatory factors, thereby aggravating disease progression in mice [112]. Interestingly, SLE activity is also related to macrophage polarization, and active SLE is more inclined to the expression of proinflammatory M1 macrophages [113]. In the subtype of M2 macrophages, we already know that M2a has the function of promoting tissue repair and fibrosis. M2b participates in immune and inflammatory responses, and M2c is involved in inactivation, remodeling, and anti-inflammatory processes [114]. Each subtype plays an individual role in SLE. In the later stages of SLE, fibrosis is a common clinical manifestation attributed to the function of macrophages, especially end-stage renal fibrosis, which is closely related to the CD206 subset of M2 macrophages, but whether it is related to M2a macrophages has not been reported [115, 116]. M2b is considered to be related to the inflammatory pathology of SLE, and the IL-10 and IFN-*γ* of M2b expressions can be detected in serum samples of SLE patients, which is associated with the deposition of immune complexes in SLE as a good inducer of M2b activation [117]. A recent animal model of SLE mice showed that blocking the Notch1 signaling pathway can hinder the polarization of M2b macrophages and improve lupus symptoms in SLE mice [118]. Therefore, selective inhibition of M2b activity can reduce its proinflammatory effect and tissue damage. There are still many unknowns about the regulation of subtypes under M2 polarization, and we need to further fill the gap. Similarly, defective M2-like macrophages exacerbate the development of SLE by uncontrollably secreting cytokines and promoting abnormal deposition of immune complexes, such as M2-like macrophages lacking heme oxygenase-1 expression found in lupus nephritis, a complication of SLE [42].

A large number of studies have found that promoting the activation of M2 macrophages and returning the M1/M2 macrophage ratio to normal level play a specific role in alleviating SLE. JAK/STAT signaling pathway is an important pathway that regulates the polarization direction of macrophages. JAK (Janus kinase) kinase family plays a crucial role in the immune system and is a series of pathological therapeutic targets, including autoimmune diseases, COVID-19-related cytokine storms, and blood cancer [119, 120]. In an in vitro experiment, it was found that peripheral blood-derived mesenchymal stem cells could secret IL-10 to induce the upregulation of JAK1/STAT3 signaling in macrophages, thereby promoting an increase in the expression of M2-type macrophages and M2-related cytokines [121]. It is known that most TLR agonists activate M1 macrophage polarization; in contrast, the toll-like receptor 2/1 agonist PAM3 can induce human monocytes to differentiate into M2-like macrophages in vitro and in vivo. The underlying mechanism involved in this process is due to PAM3 promoting monocytes differentiating into immunosuppressive macrophages by regulating the p38 MAPK and PTGS2 pathways in monocytes [122, 123]. In addition, this study also found that type I interferon can participate in the pathogenesis of SLE through the JAK/STAT pathway and is positively correlated with the development of SLE. Moreover, serine/threonine kinase AKT2 can act with IRF3 to weaken IRF3 nuclear translocation, thereby reducing the production of type I interferon. Thus, AKT2 may have a particular targeted therapeutic effect on SLE [124].

#### 3.6.3. Macrophage Polarization and Lupus Nephritis

Lupus nephritis (LN) is a common SLE complication that can lead to severe tissue damage and organ failure. The pathogenesis of LN is related to immune complex deposition, macrophage activation, and excessive release of proinflammatory cytokines. Activation of the immune complex of Fc*γ* receptors on Fc receptor-carrying cells (monocytes and macrophages) can lead to the release of inflammatory cytokines, thereby causing kidney inflammation [125]. Macrophages are the primary infiltrating cells in the kidney of LN patients and participate in the injury and repair of the kidney. Immature macrophages can be detected in the urine of patients with LN, and the frequency of those macrophages is associated with the disease progression [126].

Evidence has shown that M1 macrophages from the injured kidney have proinflammatory effects and clear apoptotic and injured cells. M2-type macrophages play a role in inhibiting inflammation and promoting tissue repair, while M2a-like macrophages are involved in fibrosis repair and progression [127], and M2c can exert anti-inflammatory and profibrotic effects [128]. Thus, changing the direction of macrophage polarization can worsen or improve the development and prognosis of LN. In most cases, alleviation of LN is mainly achieved by increasing the frequency of M2 macrophages and enhancing the anti-inflammatory properties. For example, using the pristane-induced mouse animal model, it was found that total glucosides of peony (TGP) could efficiently increase the frequency of M2 macrophages through the IL-4-mediated STAT6 signal transduction pathway and play a therapeutic role in LN through its anti-inflammatory effect [129].

The lack of Bruton's tyrosine kinase (BTK) could enhance STAT6 phosphorylation through the STAT signaling pathway, resulting in decreased M1 polarization and increased M2 polarization. In addition, the BTK inhibitor BI-BTK-1 can prevent macrophage activation by inhibiting Fc receptors and certain TLRs, decrease immune complex (IC) deposition, reduce autoantibody IgG levels, and downregulate inflammatory mediators (TNF, IL-1*β*, and IL-60) [130]. These two viewpoints are consistent and show that BTK inhibitors can promote M2 macrophage polarization, reduce the production of inflammatory factors, improve the renal microenvironment, and play an excellent protective and therapeutic role in the damaged kidney in LN. Although BTK inhibitor as a drug has successfully treated rheumatoid arthritis and multiple sclerosis in the clinic, further efforts are still needed in the clinical application in treating SLE and Sjogren's syndrome [131].

The NLRP3 inflammasome is a cytosolic protein composed of the innate immune receptor protein NLRP3, adapter protein ASC, and inflammatory protease caspase-1, playing a vital role in regulating autoimmune diseases [132]. The NLRP3 inflammasome is closely related to LN. Activation of NLRP3 inflammatory corpuscles can increase the release of proinflammatory factors, stimulate macrophages to polarize into M1, and aggravate the damage of LN. An in vitro human monocyte study found that oleamide, an endogenous fatty acid primary amide, can activate NLRP3 inflammasome, increasing cytokine IL-1*β* and macrophage polarization to proinflammatory M1 metastasis [133]. Therefore, lentivirus-mediated Fc*γ* receptor I (Fc*γ* RI) by inhibiting nuclear factors-*κ*B (NF-*κ*B) could reduce the activation of NLRP3 inflammasome, inhibit renal inflammation, and reduce the toxic effect of LN [134]. An experimental study revealed that miR-654 treatment effectively improves LN in rats by inhibiting macrophage migration inhibitory factor (MIF), selectively inhibiting ERK and AKT phosphorylation, and reducing the production of downstream inflammatory cytokines [75]. Interestingly, stimulation of human M2-like macrophages with type I interferons can lead to decreased HO-1 expression and elevated Bach1 and IL-6 expression, suggesting that dysregulated M2-like macrophages play a proinflammatory role in LN. Bach1 may be a potential therapeutic target that could restore the anti-inflammatory property of M2 macrophages [135].

#### 3.6.4. Macrophage Polarization and Rheumatoid Arthritis

Rheumatoid arthritis (RA), a commonly autoimmune disease in clinical practice, is a chronic synovial proliferative inflammation. Inflammatory changes are mainly seen in the synovial tissue of joints, cartilage, and bones, rarely in extra-articular areas such as skin and blood vessels. The prevalence rate of RA is between 0.4% and 1.3% of the population, and the prevalence rate of women is 2-3 times higher than that of men [136, 137]. To date, the etiology is still unclear. It is reported that the risk factors of RA include smoking, improper diet, exposure to ultraviolet rays, sex hormones, drugs, and periodontitis [138]. The main clinical manifestations are low fever, weight loss, joint injury, and dysfunction. When patients with RA get up early, they usually have inflexible joint activities, including morning stiffness and multiple joint symmetry involvement, often leaving joint deformities [139]. The diagnostic basis of RA includes patients' clinical symptoms, risk factor assessment, family history, and laboratory tests such as detecting biomarkers (e.g., erythrocyte sedimentation rate in serum, C-reactive protein, and RA-specific autoantibodies) [138, 140]. Clinically, the treatment of RA includes drug treatment, immune purification, surgical treatment, and patients' self-strengthening functional exercise [141, 142].

Macrophages can polarize to M1 or M2 when stimulated by different environmental factors, and the dynamic polarization process from M1 to M2 includes the presence of intermediate polarity stages. M1/M2 polarization imbalance contributes to acute or chronic RA [15, 143]. In recent years, studies have found that classically activated M1 macrophages secrete high levels of proinflammatory cytokines and chemokines and induce early inflammatory lesions in RA, and the symptoms and signs of rheumatoid arthritis (RA) are exacerbated with the increase of proinflammatory cytokines [16]. Activation of the NLRP3 inflammasome via the NF-*κ*B pathway and gasdermin family-driven phosphorylation is all related to the inflammatory process of RA [144]. In the remission of RA, the expression of the M2 macrophage (MerTK+CD206+) significantly increased, and the secretion of anti-inflammatory cytokines by M2 macrophages alleviates the symptoms and signs of RA [145]. MERTK macrophages can release lysin D1 and induce the expression of collagen genes such as COL1A to promote fibroblast repair phenotype, while the binding of MERTK to exposed phosphatidylserine (PS) on apoptotic cells further exerts phagocytosis [146, 147]. M2 macrophages, an anti-inflammatory/prorepair process, shift the disease from active to remission. Thus, in situ guided macrophage reprogramming provides valuable clues to alter the activity and severity of RA. In a mouse arthritis study, the use of M2 macrophage-derived extracellular vesicles (rich in proteins known to be involved in M2 production as well as macrophage reprogramming factors) can drive synovial macrophage polarization from the M1 type to the M2 phenotype, thereby reducing joint damage and inflammatory responses in mice [148]. In addition, we can also alleviate disease severity by directly reducing M1 and even reducing related chronic pain. For example, glaucocalyxin B (Gla B) can minimize M1 polarization in synovial macrophages by inhibiting P65 expression in the NF-*κ*B pathway [149]. Wilforlide A, an active compound in Tripterygium wilfordii Hook F, can participate in macrophage polarization through the TLR4/NF-*κ*B pathway and inhibit LPS/IFN-*γ*-induced upregulation of TLR4, which in turn inhibits NF-*κ*B activation and reduces M1 polarization [150]. As we all know, TNF can serve as a driver of RA, so anti-TNF drugs can promote M2 polarization by targeting the IL-10/STAT3 pathway [151]. Thus, these drugs play an excellent role in alleviating RA. In addition, recent studies have also shown that moxibustion has a particular therapeutic effect on RA. Moxibustion is a form of traditional Chinese medicine that mainly promotes M2 polarization through activating JAK1, JAK3, and STAT6 in the IL-4/STAT6 signaling pathway, thus reducing inflammatory cell infiltration and vasodilation, and helps alleviate the effects of RA [152]. Moreover, researchers also confirmed that sirtuin 6 (Sirt6) in bone marrow cells plays a crucial role in macrophage phenotypic switching and migration response. Sirt6 inhibits NF-*κ*B-mediated inflammatory response by interacting with the RelA subunit of NF-*κ*B, so when Sirt6 is deficient, it will promote NF-*κ*B activation and endogenous production of IL-6, thereby enhancing macrophage infiltration and M1 macrophage activation in the joint, aggravating inflammation and leading to the development and deterioration of RA [153, 154]. Macrophage polarization plays an essential role in the progression of RA. Therefore, drug regulation of macrophage repolarization may be an effective method for targeted therapy of RA. Interestingly, using a RA mouse model, plasmid DNA encoding the anti-inflammatory cytokine interleukin-10 (IL-10) pDNA and the chemotherapeutic drug betamethasone sodium phosphate (BSP) can be packaged into M2 exosomes to promote M1-to-M2 repolarization [155].

#### 3.6.5. Macrophage Polarization and Multiple Sclerosis

Multiple sclerosis (MS) is a progressive demyelinating disease of the central nervous system (CNS). It is also a tremendously challenging autoimmune disease in the clinic. At present, the etiology of MS is still unclear. Given multiple factors, MS is related to genetic and environmental factors, such as virus infection, smoking, and decreased vitamin D levels [156–158]. In accordance with the clinical course, it is mainly divided into four types: relapsing-remitting (RR), primary progressive (PP), secondary progressive (SP), and progressive-relapsing (PR), of which RRMS is the most common MS [159]. MS lesions are diffuse and multiple, the clinical manifestations of patients are complex, and different symptoms and signs occur due to the difference in lesion sites, including neuritis, limb paralysis, retrobulbar optic neuritis, mental symptoms, deafness, and vertigo [160]. Clinically, the diagnosis of MS is based on McDonald's diagnostic criteria, which mainly link the patient's clinical manifestations, magnetic resonance imaging (MRI), and brainstem auditory evoked potential and cerebrospinal fluid (CSF) examination for diagnosis [161].

MS is an immune-mediated chronic inflammatory disease, and the homeostasis of M1/M2 macrophages plays a prominent role in developing MS. In multiple sclerosis, M1 and M2 macrophages can coexist and play a dual role, playing a neuroprotective role by producing inflammatory mediators that cause nerve tissue damage and can promote growth support repair. Vogel et al. found that most foam macrophages in active MS lesions can express both M1 and M2 markers, confirming the existence of an intermediate state of macrophage activation [162]. Macrophages can form microglia within the CNS and are mainly involved in inflammation and demyelination in MS. In laboratory research, its animal model is experimental autoimmune encephalomyelitis (EAE). After activation, macrophages will release a variety of cytokines to promote the development of the disease, and M1 macrophages have a higher proinflammatory spectrum in EAE [163]. It has been found that different macrophage polarization types are involved in different stages of MS development. In the early or acute phase of MS, the polarization of microglia/macrophages to M1 promotes inflammatory damage to the nervous system. For example, circ_0000518 has been found to be elevated in MS. As the circ_0000518 RNA-binding protein, FUS can bind circ_0000518 and promote M1 macrophage polarization through the CaMKK*β*/AMPK pathway, thereby aggravating the continued progression and deterioration of MS [164].

In the late stage or recovery period of MS, microglia/macrophages polarize to M2, promote tissue repair, and reduce the severity of MS. Therefore, the treatment of MS mainly regulates macrophage polarization and cytokine levels and cytokine levels to improve the immune microenvironment. In recent years, dimethyl fumarate (DMF) has exhibited an excellent therapeutic effect on recurrent remitting MS. DMF can effectively improve the clinical score of MS patients, activate the antioxidant product of Nrf2, and reduce the tissue damage caused by ROS in MS and EAE animal models [165]. In addition, in an in vitro rat model, DMF can efficiently reduce proinflammatory mediators such as iNOS, TNF-*α*, IL-1*β*, and IL-6 synthesized by reducing ERK phosphorylation to promote M2-like macrophages [165, 166]. Moreover, the p38MAPK/SGK1 signaling pathway can promote M2 macrophage polarization and alleviate the severity of EAE in the MS model [167].

At present, biogenic amines' role in treating MS has powerfully attracted attention. Biogenic amines mainly include serotonin (5-HT), dopamine, and norepinephrine. Among them, 5-HT may regulate M2 macrophage polarization [168]. Regarding dopamine, it can directly recruit TRAF6 and its negative regulator ARRB2 as well as downstream signaling proteins such as TAK1, IKK, and PP2A through its receptor DRD5 on macrophages to form a multiprotein complex, thereby inhibiting the activation of TRAF6-mediated NF-*κ*B and the expression of proinflammatory genes, which may exert a particular inhibitory effect on macrophage polarization to M1 [169]. Meanwhile, dopamine inhibits nuclear translocation of NF-*κ*B p65 by forming dopamine quinones in microglia, thereby attenuating proinflammatory cytokine expression, a process that may be associated with reduced polarization of M1 macrophages [170]. These findings indicate that some biogenic amines can regulate macrophage polarization in MS, and researchers need to pay more attention to the molecular mechanism of macrophage polarization in MS in the future.

In addition, studies have also found that mitochondrial fission inhibitor (MDivi-1) can improve the inflammation of EAE mice, mainly by inhibiting TLR2/4 and GSK3*β*-mediated NF-*κ*B activation to promote M2 polarization [171].

#### 3.6.6. Macrophage Polarization and Guillain-Barre Syndrome

Guillain-Barre syndrome (GBS) is a peripheral nerve disease characterized by demyelinating lesions of peripheral nerves, nerve roots, and infiltration of small vascular inflammatory cells [172]. It is a relatively rare autoimmune disease. Patients with GBS often have sensory and motor disorders, such as muscle weakness, limb paralysis, and limb numbness. To date, the etiology of GBS has not been fully addressed, but in most cases, it is easy to develop after bacterial or viral infection, which is more common in men, and the incidence rate increases with age [173]. The most common animal model of GBS in scientific research is experimental autoimmune neuritis (EAN). The EAN animal model is established via immunizing Lewis rats with myelin or myelin P2 and P0 from Freund's adjuvants to develop transient paralysis [174]. The pathological manifestations of neuroedema, perivenous lymphocyte infiltration, and macrophage-mediated demyelinating are the same as GBS.

M1 and M2 macrophages can guide T cell polarization in different ways. At different stages of GBS, macrophages play either a proinflammatory or anti-inflammatory role. In the early stage of GBS, M1 macrophages promote cytotoxicity and Th1 cytokine production, leading to inflammatory damage of myelin sheath and disease development [175]; in the late stage of GBS, M2 macrophages promote Th2 immune response and the secretion of anti-inflammatory cytokines and participate in the recovery of disease and the repair of the myelin sheath and axons [176]. M1 macrophage-derived exosomes can exacerbate EAN by enhancing the Th1 and Th17 responses, while M2 macrophage-derived exosomes reduce disease severity [177].

The Notch signaling pathway is an important pathway for macrophage polarization. The Notch receptor family consists of 4 members (Notch1-4), the ligand family consists of 5 members (Delta1, Delta3, Delta4, Jagged1, and Jagged2), and NICD and RBP-J as the downstream molecules of the Notch signaling pathway are also actively involved in the regulation of M1 macrophage polarization [178]. Oridonin (a herbal extract compound) may downregulate the expression of Notch1, Jagged-2, and downstream molecules by blocking the Notch pathway, promoting the transfer of M1 to M2, leading to the reduction of proinflammatory cytokines, and significantly improving the progression of EAN [179]. The NF-*κ*B signaling is also an effective target for the treatment of EAN. Thus, reducing the polarization of M1 macrophages and promoting the polarization of M2 by inhibiting p65 phosphorylation in the NF-*κ*B pathway can alleviate EAN [180].

#### 3.6.7. Macrophage Polarization and Autoimmune Uveitis

Uveitis is an inflammatory disease of the iris, ciliary body, and choroid tissue in the eye. Clinically, approximately 35% of uveitis patients have a severe visual impairment or even blindness. The etiology of uveitis is complex and can be divided into infectious or noninfectious uveitis. Many studies have confirmed that noninfectious uveitis is mainly related to autoimmunity, that is, the deposition of antigen-antibody complexes in the capillary-rich uvea. It is reported that uveitis primarily occurs in young people. Currently, the main treatment methods include the local or systemic application of glucocorticoids and ciliary muscle paralysis [181].

Experimental autoimmune uveitis (EAU) is an ideal animal model of human autoimmune uveitis. EAU model induction is the immunization of susceptible rodents using proteins or peptides extracted from the retina, iris, or ciliary body. This process is combined with complete Freund's adjuvant and tuberculin. Mice are more accessible to transgene and propagation than other animals, and the eye structure of mice is similar to that of human beings, so most of the research in recent years has used EAU mouse animal models [174, 182]. Macrophages participate in the whole process of EAU and play different roles in different stages of the development of EAU [183]. It is well known that when macrophage M1/M2 polarization is unbalanced, it will affect the differentiation of Th cells, leading to the imbalanced Th1/Th2 and Th17/Treg ratios. Th1 and Th17 reactions can aggravate inflammation-related pathogenicity, whereas Th2 and regulatory T (Treg) reactions can alleviate the process of EAU [184]. The Notch signaling pathway plays a key role in the pathogenesis of EAU. It has been confirmed that the expression of Notch1, DLL4, IL-10, IL-17, ROR*γ*t, and Foxp3 is elevated in the pathogenesis of EAU, and increased polarization of M1 macrophages and an imbalance in the ratio of Th17/Treg occur [185]. Similarly, using an EAU rat model, it is found that Longdan Xiegan decoction (LXD), a traditional Chinese medicine compound, can effectively decrease the expression of Notch 1 and Delta4, inhibit the activation of the Notch pathway, and reduce the expression of IL-17 to alleviate the ocular inflammatory reaction and effectively improve the intraocular immune microenvironment [186, 187]. Therefore, the use of Notch signaling inhibitor DAPT can inhibit M1 macrophage polarization and reduce Th17 cell response, thereby leading to the restoration of the Th17/Treg ratio.

The NF-*κ*B signaling pathway also plays an essential role in the pathogenic mechanism of EAU. In mammals, the NF-*κ*B family consists of five members, including RelA (p65), RelB, c-Rel, NF-*κ*B1 (p50), and NF-*κ*B2 (p52), which form various dimer complexes that regulate gene transcription by binding to 10bp-specific sequences (-*κ*B sites) on target genes [188]. Recent studies have found that galactose lectin-3 is expressed in EAU and has a particular proinflammatory effect, and TD139 (galactose lectin-3 inhibitor) can inhibit the activation of NF-*κ*B P65 by downregulating the expression of TLR4/MyD88, thereby reducing M1 polarization and contributing to the treatment of EAU [189]. Similarly, IMD-0354, an inhibitor of IKK*β*, can also minimize Th1/Th17-mediated inflammation by inhibiting NF-*κ*B p65 in an animal model of EAU [190].

In addition to the Notch and NF-*κ*B signaling pathways described above, PI3K/AKT/FOXO1 phosphorylation is also considered novel pathogenesis of EAU, and phosphodiesterase-4 inhibitors (apremilast, PDE4i) can reduce the Th1 and Th17 frequencies by inhibiting the downstream transcription factor FOXO1 expression in the PI3K/AKT pathway and enhancing the Treg cell response to alleviate EAU [191]. Interestingly, ICA combined with peroxidase-3 (PRDx3) can downregulate H2O2 and activate the GPX4/SLC7A11/ACSl4 pathway, which may regulate the transfer of macrophage polarization from M1 to M2, exhibiting a specific therapeutic potential for EAU [192].

#### 3.6.8. Macrophage Polarization and Sjogren's Syndrome

Sjogren's syndrome (SS) is a chronic autoimmune disease commonly occurring in middle-aged women. Clinically, SS can be divided into primary and secondary SS. Primary SS refers to the separate onset of SS, and secondary SS is mainly induced by SLE and RA. The clinical manifestations of SS are diverse and can involve the whole-body system and specific target organs [193]. It is mainly due to the abnormal function of lacrimal glands and salivary glands, leading to dry skin and mucosa. Currently, the clinical diagnosis largely depends on patients' physical signs, pathological biopsy, imaging examination, and detection of autoantibodies.

The etiology of SS is still unclear, but sustained B-cell activation and proliferation of Th1 and Th17 cells contribute to disease progression [194]. IFN-induced gene overexpression has been found in patients with SS, including interferon-induced protein 44 (IFI44) and transporter 2 ATP-binding cassette (TAP2) [195, 196]. Moreover, there is an interaction between IFN and B lymphocyte activation, and B cells can induce the production of IFN, which in turn facilitates the production of autoantibodies [79, 195]. Therefore, the pathogenesis of SS in the innate immune system is closely related to the presence of type I interferon [197]. In adaptive immunity, B cells and T cells are activated by type I and II interferons. Adaptive immunity involves B cell activation to produce antibody and T cell polarization, in which Th1 and Th17 proportions will increase; meanwhile, Treg cells are also involved in this process [194, 195]. These findings remind us that the interference strategy against IFN may be effective for treating SS.

After macrophage polarization, both macrophage subtypes exist in patients with primary Sjogren's syndrome. M1 macrophages exist in the early stage of PSS, which generate inflammatory factors such as TNF-*α*, IL-6, IL-1*β*, and IL-12 to play a proinflammatory role and further activate CD4^+^ T cells to differentiate into Th1 cell lineage, leading to the occurrence and development of submandibular gland inflammation [198]. In an in vitro experiment based on a rabbit animal model, M2 macrophages secrete IL-10 and TGF-*β* and other anti-inflammatory mediators, which contribute to the regression of inflammation and tissue regeneration, and alleviate autoimmune lacrimal gland inflammation, thereby playing an anti-inflammatory role [199]. When SS develops to the late stage of the disease, the chronic inflammation proceeds to irreversible salivary gland fibrosis, which is mainly mediated by M2 macrophages. The TGF-*β* signal transduction pathways that induce fibrosis are divided into SMAD regulation and non-SMAD regulation. TGF-*β* can promote M2 macrophage polarization by activating SMAD2/3/4 trimer complexes, and this pathway can also promote fibroblast-to-myofibroblast transformation [200]. Non-SMAD pathways can activate the MAPK/RAS signaling pathway. RREB1, a molecular junction between RAS and TGF-*β* pathways, can also induce development and fibrosis [201–203]. It has been confirmed that multiple pathways and signaling molecules are involved in the pathogenesis of PSS inflammatory response. For example, researchers have found that metformin could reduce mTOR by inhibiting the activation of 5′ adenosine monophosphate-activated protein kinase (AMPK), reduce the production of antibodies after STAT3 phosphorylation of B cells, promote T cell differentiation into Treg, enhance anti-inflammatory immunity, and thus improve salivary gland function, suggesting that mTOR may be a promising therapeutic target [204]. In addition, IL-21 will increase PSS, which can induce the phosphorylation of STAT1 and STAT3 through the JAK/STAT pathway and promote the proliferation of Th17 cells, thereby playing a certain role in the pathogenesis and treatment of PSS [205]. Other studies have revealed that activation of mTOR can induce Th17 differentiation and inhibit the Treg effect through the PI3K/AKT pathway, and Th17/Treg imbalance aggravates inflammation and induces apoptosis [205]. Nevertheless, other researchers confirmed that PI3K/AKT can also alleviate SS symptoms. HUC MSCs can promote M2 macrophage polarization by activating PI3K/AKT pathway, thereby inhibiting the inflammatory response of autoimmune lacrimal gland inflammation [199]. In addition, TLR2 and TLR4 expression is found to be increased in primary Sjogren's syndrome, and MAPK and NF-*κ*B are activated by MyD88, which induces M1 macrophage polarization to secrete inflammatory factors. Therefore, blocking this pathway has a particular therapeutic effect on SS [206, 207].

The role of macrophage polarization balance in the pathogenesis of SS cannot be ignored. Still, in recent years, the research on SS has mainly focused on the pathogenesis of epithelial cells. Hence, the relationship between macrophage polarization and the molecular mechanism of SS remains to be further explored.

#### 3.6.9. Macrophage Polarization and Systemic Sclerosis

Systemic sclerosis (SSc) is an autoimmune disease in which chronic progressive inflammation and fibrosis of tissues and organs are the main lesions after excessive extracellular matrix production [208]. The pathogenesis of SSc is unknown, but its pathogenesis involves activating various immune cells, including macrophages. The disease may involve proinflammatory M1 macrophages and profibrotic M2 macrophages with activation copathogenic disease [209–211]. Patients with SSc are predominantly inflammatory lesions in the early stage, followed by extensive fibrosis, cytoskeletal rearrangement, ECM remodeling, increased type I collagen, fibronectin, and *α*-SMA, including FN1 (the gene encoding fibronectin) expression and TGF-*β* signaling pathway [212]. After TGF-*β* activation, macrophages and fibroblasts can activate each other, further increasing tissue thickness and hardness and mediating fibrosis [213]. Various studies are currently aimed at regulating macrophage polarization to improve SSc symptoms. The adenosine deaminase of RNA can promote M1 macrophage activation at the beginning of SSc and control the release of inflammatory mediators (iNOS, IL-*β*) by regulating the NF-*κ*B signaling pathway, so ADAR1 deficiency in macrophages can significantly improve skin and lung sclerosis [214]. In addition, methyl-CpG-binding domain 2 (Mbd2) selectively binds to the SH2-containing inositol 5′-phosphatase (Ship) promoter in macrophages and inhibits Ship expression, thereby inhibiting PI3K/AKT signaling and suppressing M2 macrophages, so exogenous delivery of Mbd2 can protect mouse models from fibrosis damage [215]. Therefore, the ideal drug should be able to block the macrophage polarization pathway and reduce the activated macrophage to achieve combined anti-inflammatory and antifibrotic effects. Still, the current research mainly focuses on regulating a macrophage, which has certain limitations in disease treatment. It may be that the future development of drugs that jointly inhibit M1 and M2 macrophages is a potential route to be explored.

**Figure 1:**
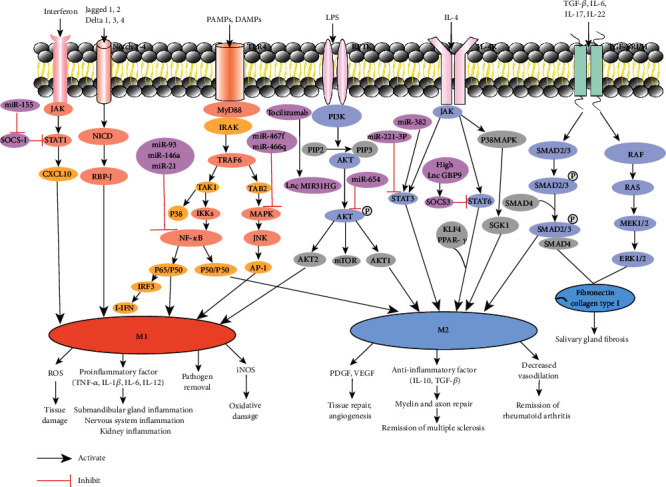
A schematic illustration of the relationship between macrophage polarization-associated signaling pathways and autoimmune diseases.

Taken together, macrophage polarization plays an essential role in autoimmune diseases. In addition to the above-mentioned autoimmune diseases, macrophage polarization imbalance also occurs in ulcerative colitis, nonalcoholic liver disease, autoimmune diabetes, and other autoimmune diseases [216–218]. In the process of driving the polarization of M1 and M2 macrophages, there are many signaling pathways involved, including the JAK-STAT, MAPK, TGF-*β*/SMAD, Notch, and PI3K-AKT pathways (Figure 1). Therefore, a deep understanding of the mechanism of macrophage polarization in the occurrence and development of autoimmune diseases can provide new insight into clinical treatment of autoimmune diseases by regulating macrophage polarization balance.

## 4. Conclusion

In conclusion, macrophages are a class of cells with complex functions in the immune system. Macrophages can be mainly polarized into M1 and M2 macrophages according to the changes in the microenvironmental conditions in which they are located, and M1 macrophages promote the development of inflammation, accelerate extracellular matrix degradation and apoptosis, and regulate and promote the Th1 type immune response; while M2 macrophages inhibit the proliferation and activation of T cells, regulate the Th2 immune response, and aid in tissue remodeling. Macrophage polarization plays an essential role in autoimmune diseases and reflects great complexity, macrophages are mostly manifested as proinflammatory M1 in the early stage of disease repair, and the M2 type that promotes healing is common in the late stage of disease repair. In addition to polarization into M1 and M2 states, it is possible to change from proinflammatory (M1) to prohealing (M2) phenotypes during tissue repair. Moreover, autoimmune diseases can share M1 and M2 phenotypes and have an intermediate polarization state. The imbalance of M1/M2 polarization plays a vital role in autoimmune diseases. Therefore, regulating macrophage polarization's direction can improve autoimmune disease pathogenesis. Presently, the therapeutic strategy of finding therapeutic targets for autoimmune diseases concentrated on regulating macrophage polarization has indeed made significant progress. However, most of the results are based on the data obtained from animal models, and there may be some species differences between animal models and human beings. Therefore, a more comprehensive understanding of the relationship between macrophage polarization and the occurrence and development of autoimmune diseases can find more effective therapeutic targets for autoimmune diseases. It is also the focus of people's efforts in the future.

## Figures and Tables

**Table 1:** Phenotypes, stimulants, biomarkers, secretions, and functions of macrophages.

Macrophage phenotype	Stimulants	Biomarkers	Secretions	Functions	Ref.
M1 (classically activated macrophages)	IFN-*γ*, LPS, TNF-*α*, GM-CSF	CD86, CD40, CD38, NF-*κ*B, STAT1	TNF-*α*, IL-1*α*, IL-12, IL-23, IL-1*β*, IL-6, ROS, and RNS	Promote Th1 immune response, promote inflammatory response, fight pathogens, and inhibit the occurrence and development of tumors	[7, 8, 42–45]
M2a (wound healing macrophages)	IL-4, IL-13, M-CSF	CD206, IL-1R, CCL17, Fizz1, STAT6	TGF-*β*, IL-10, insulin-like growth factor (IGF), and fibronectin	Promote tissue repair and remodeling, promote fibrosis, and promote type II immune response by enhancing polyamines, collagen synthesis	[45, 53–56]
M2b (regulatory macrophages)	Immune complex, TLR agonist, IL-1R agonist	IL-10, CCL1, LIGHT, CD86, SPHK1, TNF-*α*, IL-6, ERK, AP-1	Proinflamatory cytokines (IL-1*β*, IL-6, and TNF-*α*), anti-inflammatory cytokine (IL-10 and low levels of IL-12)	Involve in proinflammatory and anti-inflammatory responses, immunomodulation, and Th2 activation	[45, 54–58]
M2c (acquired inactivated macrophages)	Glucocorticoids, TGF-*β*, IL-10	CD163, Mer receptor tyrosine kinase (MerTK), STAT3	IL-10, TGF-*β*	Immune tolerance and tissue repair, suppress inflammation, promote phagocytosis and cholesterol efflux	[45, 55, 56, 58, 59]
M2d (tumor-associated macrophages	TLR, adenosine A2A receptor *γ*, IL-6	Vascular endothelial growth factor A (VEGF-A), HIF-1*α*	Proteolytic enzymes (MMP-2), growth factors (VEGF), and anti-inflammatory mediators (TGF-*β*)	Beneficial for angiogenesis and tumor metastasis	[45, 55, 56, 60–64]

**Table 2:** MicroRNAs involved in regulating macrophage polarization.

MicroRNA	Organism	Cell	Target	Function	Ref.
miR-155	Human	Monocytes	SOCS1, IL-13R*α*1, C/EBP-*β*	Activates STAT1 and inhibits STAT6, thereby promoting macrophage polarization	[67]
miR-146a	Human	Peripheral blood mononuclear cells	TRAF6, IRAK1, IRAK2, IRF3	Limits NF-*κ*B and IRF3 and inhibits M1 macrophage polarization	[68]
miR-let-7a	Human	Macrophages	HMGA2	IRF5 is inhibited by the PI3K pathway, thereby inhibiting M1 macrophage polarization	[69]
miR-33	Mouse	Monocytes	AMP activates protein kinases (AMPK)	Promotes M2 macrophage polarization, elevates Treg cells	[70]
miR-223	Mouse	Macrophages	Pknox1, Sp3	Promotes M2 macrophage polarization, inhibits NLPR3 inflammasome	[71]
miR-21	Human	Macrophages	PTEN, PDCD4	Promotes M2 macrophage polarization, decreases NF-*κ*B signaling, and increases IL-10 production	[72]
miR-125a	Human	Macrophages	FIH1, IRF4	Promotes M1 macrophage polarization	[73]
miR-100-5p	Human	Mesenchymal stem cells	Unknown	Promotes M2 macrophage polarization by regulating the PI3K-AKT pathway	[74]
miR-654	Human	Macrophages	MIF	Reduces macrophage downstream proinflammatory cytokines by inhibiting phosphorylation of ERK and AKT	[75]
miR-382	Mouse	Tubular epithelial cells	SIRP-*α*	Activates the STAT3 signaling pathway to promote M2 macrophage polarization	[76]
miR-221-3p	Human	Macrophages	JAK3	Inhibits the JAK3/STAT3 pathway to promote M1 macrophage polarization	[77]
miR-467f	Mouse	Macrophages	Map3k8, Mk2	Inhibits M1 macrophage polarization	[78]
miR-93	Rat	Macrophages	IRAK4	Inhibits NF-*κ*B activation and negatively regulates M1 macrophage polarization	[79]

**Table 3:** lncRNAs involved in regulating macrophage polarization.

lncRNA	Organism	Cell	Target	Function	Ref.
lncRNA GAS5	Human	Macrophages	CCL1	Promotes M2 macrophage polarization	[80]
lncRNA RN7SK	Human	Macrophages	P-TEFb	Promotes M2 macrophage polarization, negatively regulates antigen uptake/processing and bacterial phagocytosis	[80]
lncRNA ANCR	Mouse	Macrophages	FOXO1	Reduces the level of IL-6 and IL-1*β* in cells, inhibits M1 macrophage polarization	[81]
lncRNA Mirt2	Mouse	Macrophages	TRAF6	Inhibits the activation of NF-*κ*B and MAPK pathways and inhibits M1 macrophage polarization	[82]
lncRNA FAO	Mouse	Macrophages	HADHB subunit	Promotes M2 macrophage polarization, inhibits proinflammatory cytokines	[83]
lncRNA GBP9	Mouse	Bone marrow-derived macrophages	SOCS3	Inhibits the STAT6 pathway, promotes M1 macrophage polarization	[84]
lncRNA GAS5	Mouse	Microglia	TRF2	Induces the polarization of macrophages to M2	[85]
lncRNA260	Rat	Macrophages	Interleukin-28 receptor *α*	Activates the JAK-STAT and PI3K/AKT signaling pathways to promote M2 macrophage polarization	[86]

## References

[1] Bieber K., Hundt J. E., Yu X. (2023). Autoimmune pre-disease. *Autoimmunity Reviews*.

[2] Committee for the Assessment of NIH Research on Autoimmune Diseases; Board on Population Health and Public Health Practice; Health and Medicine Division; National Academies of Sciences, Engineering, and Medicine (2022). *Enhancing NIH Research on Autoimmune Disease*.

[3] Glover K., Mishra D., Singh T. R. R. (2021). Epidemiology of ocular manifestations in autoimmune disease. *Frontiers in Immunology*.

[4] Ren P., Lu L., Cai S., Chen J., Lin W., Han F. (2021). Alternative splicing: a new cause and potential therapeutic target in autoimmune disease. *Frontiers in Immunology*.

[5] Kolliniati O., Ieronymaki E., Vergadi E., Tsatsanis C. (2022). Metabolic regulation of macrophage activation. *Journal of Innate Immunity*.

[6] Wynn T. A., Vannella K. M. (2016). Macrophages in tissue repair, regeneration, and fibrosis. *Immunity*.

[7] Wang N., Liang H., Zen K. (2014). Molecular mechanisms that influence the macrophage M1-M2 polarization balance. *Frontiers in Immunology*.

[8] Sun Y., Li J., Xie X. (2021). Macrophage-osteoclast associations: origin, polarization, and subgroups. *Frontiers in Immunology*.

[9] Boutilier A. J., Elsawa S. F. (2021). Macrophage polarization states in the tumor microenvironment. *International Journal of Molecular Sciences*.

[10] van der Heide D., Weiskirchen R., Bansal R. (2019). Therapeutic targeting of hepatic macrophages for the treatment of liver diseases. *Frontiers in Immunology*.

[11] Chung E. J., Kwon S., Shankavaram U., White A. O., Das S., Citrin D. E. (2022). Natural variation in macrophage polarization and function impact pneumocyte senescence and susceptibility to fibrosis. *Aging*.

[12] Henderson N. C., Rieder F., Wynn T. A. (2020). Fibrosis: from mechanisms to medicines. *Nature*.

[13] Nakagawa M., Karim M. R., Izawa T., Kuwamura M., Yamate J. (2021). Immunophenotypical characterization of M1/M2 macrophages and lymphocytes in cisplatin-induced rat progressive renal fibrosis. *Cells*.

[14] Duan J., Liu X., Wang H., Guo S. W. (2018). The M2a macrophage subset may be critically involved in the fibrogenesis of endometriosis in mice. *Reproductive Biomedicine Online*.

[15] Cutolo M., Campitiello R., Gotelli E., Soldano S. (2022). The role of M1/M2 macrophage polarization in rheumatoid arthritis synovitis. *Frontiers in Immunology*.

[16] Wang Y., Han C. C., Cui D., Li Y., Ma Y., Wei W. (2017). Is macrophage polarization important in rheumatoid arthritis?. *International Immunopharmacology*.

[17] Tardito S., Martinelli G., Soldano S. (2019). Macrophage M1/M2 polarization and rheumatoid arthritis: a systematic review. *Autoimmunity Reviews*.

[18] Zhang W., Zhou Q., Xu W. (2013). DNA-dependent activator of interferon-regulatory factors (DAI) promotes lupus nephritis by activating the calcium pathway. *The Journal of Biological Chemistry*.

[19] Ishiguro N., Moriyama M., Furusho K. (2020). Activated M2 macrophages contribute to the pathogenesis of IgG4-related disease via toll-like receptor 7/interleukin-33 signaling. *Arthritis & Rhematology*.

[20] Yona S., Gordon S. (2015). From the reticuloendothelial to mononuclear phagocyte system - the unaccounted years. *Frontiers in Immunology*.

[21] Mertens C., Marques O., Horvat N. K., Simonetti M., Muckenthaler M. U., Jung M. (2021). The macrophage iron signature in health and disease. *International Journal of Molecular Sciences*.

[22] Perdiguero E. G., Geissmann F. (2016). The development and maintenance of resident macrophages. *Nature Immunology*.

[23] Yang J., Zhang L., Yu C., Yang X. F., Wang H. (2014). Monocyte and macrophage differentiation: circulation inflammatory monocyte as biomarker for inflammatory diseases. *Biomarker Research*.

[24] Naito M., Umeda S., Yamamoto T. (1996). Development, differentiation, and phenotypic heterogeneity of murine tissue macrophages. *Journal of Leukocyte Biology*.

[25] Davies L. C., Jenkins S. J., Allen J. E., Taylor P. R. (2013). Tissue-resident macrophages. *Nature Immunology*.

[26] Wynn T. A., Chawla A., Pollard J. W. (2013). Macrophage biology in development, homeostasis and disease. *Nature*.

[27] Ruytinx P., Proost P., Van Damme J., Struyf S. (2018). Chemokine-induced macrophage polarization in inflammatory conditions. *Frontiers in Immunology*.

[28] Chen Y., Zhang X. (2017). Pivotal regulators of tissue homeostasis and cancer: macrophages. *Experimental Hematology & Oncology*.

[29] Mesquida-Veny F., Del Río J. A., Hervera A. (2021). Macrophagic and microglial complexity after neuronal injury. *Progress in Neurobiology*.

[30] Zabala A., Vazquez-Villoldo N., Rissiek B. (2018). P2X4 receptor controls microglia activation and favors remyelination in autoimmune encephalitis. *EMBO Molecular Medicine*.

[31] Thapa B., Lee K. (2019). Metabolic influence on macrophage polarization and pathogenesis. *BMB Reports*.

[32] Viola A., Munari F., Sánchez-Rodríguez R., Scolaro T., Castegna A. (2019). The metabolic signature of macrophage responses. *Frontiers in Immunology*.

[33] Kieler M., Hofmann M., Schabbauer G. (2021). More than just protein building blocks: how amino acids and related metabolic pathways fuel macrophage polarization. *The FEBS Journal*.

[34] Wang Y., Li N., Zhang X., Horng T. (2021). Mitochondrial metabolism regulates macrophage biology. *The Journal of Biological Chemistry*.

[35] Corcoran S. E., O'Neill L. A. J. (2016). HIF1*α* and metabolic reprogramming in inflammation. *The Journal of Clinical Investigation*.

[36] Yu X. H., Zhang D. W., Zheng X. L., Tang C. K. (2019). Itaconate: an emerging determinant of inflammation in activated macrophages. *Immunology and Cell Biology*.

[37] Peace C. G., O'Neill L. A. (2022). The role of itaconate in host defense and inflammation. *The Journal of Clinical Investigation*.

[38] O’Neill L. A., Artyomov M. N. (2019). Itaconate: the poster child of metabolic reprogramming in macrophage function. *Nature Reviews Immunology*.

[39] Mosser D. M., Edwards J. P. (2008). Exploring the full spectrum of macrophage activation. *Nature Reviews Immunology*.

[40] Murray P. J., Allen J. E., Biswas S. K. (2014). Macrophage activation and polarization: nomenclature and experimental guidelines. *Immunity*.

[41] Cho H., Kwon H. Y., Lee S. H., Lee H. G., Kang N. Y., Chang Y. T. (2023). Development of a fluorescent probe for M2 macrophages *via* gating-oriented live-cell distinction. *Journal of the American Chemical Society*.

[42] Ahamada M. M., Jia Y., Wu X. (2021). Macrophage polarization and plasticity in systemic lupus erythematosus. *Frontiers in Immunology*.

[43] Murray P. J., Wynn T. A. (2011). Obstacles and opportunities for understanding macrophage polarization. *Journal of Leukocyte Biology*.

[44] Locati M., Curtale G., Mantovani A. (2020). Diversity, mechanisms, and significance of macrophage plasticity. *Annual Review of Pathology*.

[45] Avila-Ponce de León U., Vázquez-Jiménez A., Matadamas-Guzman M., Pelayo R., Resendis-Antonio O. (2021). Transcriptional and microenvironmental landscape of macrophage transition in cancer: a Boolean analysis. *Frontiers in Immunology*.

[46] Funes S. C., Rios M., Escobar-Vera J., Kalergis A. M. (2018). Implications of macrophage polarization in autoimmunity. *Immunology*.

[47] Ross E. A., Devitt A., Johnson J. R. (2021). Macrophages: the good, the bad, and the gluttony. *Frontiers in Immunology*.

[48] Ying W., Cheruku P. S., Bazer F. W., Safe S. H., Zhou B. (2013). Investigation of macrophage polarization using bone marrow derived macrophages. *Journal of Visualized Experiments*.

[49] Tariq M., Zhang J. Q., Liang G. K., He Q. J., Ding L., Yang B. (2017). Gefitinib inhibits M2-like polarization of tumor-associated macrophages in Lewis lung cancer by targeting the STAT6 signaling pathway. *Acta Pharmacologica Sinica*.

[50] Harwani S. C. (2018). Macrophages under pressure: the role of macrophage polarization in hypertension. *Translational Research*.

[51] Wang C., Ma C., Gong L. (2021). Macrophage polarization and its role in liver disease. *Frontiers in Immunology*.

[52] Mosser D. M., Hamidzadeh K., Goncalves R. (2021). Macrophages and the maintenance of homeostasis. *Cellular & Molecular Immunology*.

[53] Mantovani A., Sica A., Sozzani S., Allavena P., Vecchi A., Locati M. (2004). The chemokine system in diverse forms of macrophage activation and polarization. *Trends in Immunology*.

[54] Wang L. X., Zhang S. X., Wu H. J., Rong X. L., Guo J. (2019). M2b macrophage polarization and its roles in diseases. *Journal of Leukocyte Biology*.

[55] Rojas J., Salazar J., Martínez M. S. (2015). Macrophage heterogeneity and plasticity: impact of macrophage biomarkers on atherosclerosis. *Scientifica*.

[56] Abdelaziz M. H., Abdelwahab S. F., Wan J. (2020). Alternatively activated macrophages; a double-edged sword in allergic asthma. *Journal of Translational Medicine*.

[57] Yang R., Liao Y., Wang L. (2019). Exosomes derived from M2b macrophages attenuate DSS-induced colitis. *Frontiers in Immunology*.

[58] Junior L. Y. S., Nguyen H. T., Salmanida F. P., Chang K. T. (2021). MERTK+/hi M2c macrophages induced by baicalin alleviate non-alcoholic fatty liver disease. *International Journal of Molecular Sciences*.

[59] Yang P., Zhang X., Lin Z. (2020). Adoptive transfer of polarized M2c macrophages ameliorates acute rejection in rat liver transplantation. *American Journal of Translational Research*.

[60] Arora S., Dev K., Agarwal B., Das P., Syed M. A. (2018). Macrophages: their role, activation and polarization in pulmonary diseases. *Immunobiology*.

[61] Wang Q., Ni H., Lan L., Wei X., Xiang R., Wang Y. (2010). Fra-1 protooncogene regulates IL-6 expression in macrophages and promotes the generation of M2d macrophages. *Cell Research*.

[62] Orecchioni M., Ghosheh Y., Pramod A. B., Ley K. (2019). Macrophage polarization: different gene signatures in M1(LPS+) vs. classically and M2(LPS-) vs. Alternatively Activated Macrophages. *Frontiers in Immunology*.

[63] Lü W. D., Liu Y. Z., Yang Y. Q. (2022). Effect of naturally derived surgical hemostatic materials on the proliferation of A549 human lung adenocarcinoma cells. *Materials Today Bio*.

[64] Hou Y., Shi J., Guo Y., Shi G. (2022). Inhibition of angiogenetic macrophages reduces disc degeneration-associated pain. *Frontiers in Bioengineering and Biotechnology*.

[65] Anders C. B., Lawton T. M. W., Smith H. L., Garret J., Doucette M. M., Ammons M. C. B. (2022). Use of integrated metabolomics, transcriptomics, and signal protein profile to characterize the effector function and associated metabotype of polarized macrophage phenotypes. *Journal of Leukocyte Biology*.

[66] Matsui M., Corey D. R. (2017). Non-coding RNAs as drug targets. *Nature Reviews Drug Discovery*.

[67] Li H., Jiang T., Li M. Q., Zheng X. L., Zhao G. J. (2018). Transcriptional regulation of macrophages polarization by MicroRNAs. *Frontiers in Immunology*.

[68] Niu X., Schulert G. S. (2019). Functional regulation of macrophage phenotypes by microRNAs in inflammatory arthritis. *Frontiers in Immunology*.

[69] Zhu W., Yu J., Qiu S. (2017). MiR-let-7a regulates anti-citrullinated protein antibody-induced macrophage activation and correlates with the development of experimental rheumatoid arthritis. *International Immunopharmacology*.

[70] Ouimet M., Ediriweera H. N., Gundra U. M. (2015). MicroRNA-33-dependent regulation of macrophage metabolism directs immune cell polarization in atherosclerosis. *The Journal of Clinical Investigation*.

[71] Nguyen M. A., Hoang H. D., Rasheed A. (2022). miR-223 exerts translational control of proatherogenic genes in macrophages. *Circulation Research*.

[72] Meng F., Henson R., Wehbe-Janek H., Ghoshal K., Jacob S. T., Patel T. (2007). MicroRNA-21 regulates expression of the PTEN tumor suppressor gene in human hepatocellular cancer. *Gastroenterology*.

[73] Zhao J. L., Huang F., He F. (2016). Forced activation of notch in macrophages represses tumor growth by upregulating miR-125a and disabling tumor-associated macrophages. *Cancer Research*.

[74] Li K., Yan G., Huang H. (2022). Anti-inflammatory and immunomodulatory effects of the extracellular vesicles derived from human umbilical cord mesenchymal stem cells on osteoarthritis via M2 macrophages. *Journal of Nanobiotechnology*.

[75] Tu Y., Guo R., Li J. (2019). MiRNA regulation of MIF in SLE and attenuation of murine lupus nephritis with miR-654. *Frontiers in Immunology*.

[76] Wang X., Jia P., Ren T. (2022). MicroRNA-382 promotes M2-like macrophage via the SIRP-*α*/STAT3 signaling pathway in aristolochic acid-induced renal fibrosis. *Frontiers in Immunology*.

[77] Quero L., Tiaden A. N., Hanser E. (2020). miR-221-3p drives the shift of M2-macrophages to a pro-inflammatory function by suppressing JAK3/STAT3 activation. *Frontiers in Immunology*.

[78] Giunti D., Marini C., Parodi B. (2021). Role of miRNAs shuttled by mesenchymal stem cell-derived small extracellular vesicles in modulating neuroinflammation. *Scientific Reports*.

[79] Negrini S., Emmi G., Greco M. (2022). Sjögren's syndrome: a systemic autoimmune disease. *Clinical and Experimental Medicine*.

[80] Ahmad I., Valverde A., Naqvi R. A., Naqvi A. R. (2020). Long non-coding RNAs RN7SK and GAS5 regulate macrophage polarization and innate immune responses. *Frontiers in Immunology*.

[81] Xie C., Guo Y., Lou S. (2020). lncRNA ANCR promotes invasion and migration of gastric cancer by regulating FoxO1 expression to inhibit macrophage M1 polarization. *Digestive Diseases and Sciences*.

[82] Du M., Yuan L., Tan X. (2017). The LPS-inducible lncRNA Mirt2 is a negative regulator of inflammation. *Nature Communications*.

[83] Nakayama Y., Fujiu K., Yuki R. (2020). A long noncoding RNA regulates inflammation resolution by mouse macrophages through fatty acid oxidation activation. *Proceedings of the National Academy of Sciences of the United States of America*.

[84] Zhou J., Li Z., Wu T., Zhao Q., Zhao Q., Cao Y. (2020). LncGBP9/miR-34a axis drives macrophages toward a phenotype conducive for spinal cord injury repair via STAT1/STAT6 and SOCS3. *Journal of Neuroinflammation*.

[85] Sun D., Yu Z., Fang X. (2017). lncRNA GAS5 inhibits microglial M2 polarization and exacerbates demyelination. *EMBO Reports*.

[86] Yang X. X., Li Y. Y., Gong G., Geng H. Y. (2022). lncRNA260 siRNA accelerates M2 macrophage polarization and alleviates oxidative stress via inhibiting IL28RA gene alternative splicing. *Oxidative Medicine and Cellular Longevity*.

[87] Li H., Luo F., Jiang X. (2022). CircITGB6 promotes ovarian cancer cisplatin resistance by resetting tumor-associated macrophage polarization toward the M2 phenotype. *Journal for Immunotherapy of Cancer*.

[88] Huang X., Wang J., Guan J. (2022). Exosomal Circsafb2 reshaping tumor environment to promote renal cell carcinoma progression by mediating M2 macrophage polarization. *Frontiers in Oncology*.

[89] Fu H., Chu L., Yuan Y. S., Liao S., Wang G. H. (2022). Circular RNA ACTR2 activates M2 polarization of macrophages through activating yes-associated protein signalling and contributes to renal fibrosis. *Immunology*.

[90] Liu C. H., Lu Y. L., Huang H. T. (2021). Association of lncRNA-GAS5 gene polymorphisms and PBMC LncRNA-GAS5 level with risk of systemic lupus erythematosus in Chinese population. *Journal of Cellular and Molecular Medicine*.

[91] Taheri M., Eghtedarian R., Dinger M. E., Ghafouri-Fard S. (2020). Exploring the role of non-coding RNAs in the pathophysiology of systemic lupus erythematosus. *Biomolecules*.

[92] Zhang F., Wu L., Qian J. (2016). Identification of the long noncoding RNA NEAT1 as a novel inflammatory regulator acting through MAPK pathway in human lupus. *Journal of Autoimmunity*.

[93] Cao L., Jiang H., Yang J. (2021). LncRNA MIR31HG is induced by tocilizumab and ameliorates rheumatoid arthritis fibroblast-like synoviocyte-mediated inflammation via miR-214-PTEN-AKT signaling pathway. *Aging*.

[94] Hsu Y. R., Chang S. W., Lin Y. C., Yang C. H. (2015). Expression of microRNAs in the eyes of Lewis rats with experimental autoimmune anterior uveitis. *Mediators of Inflammation*.

[95] Sun Y., Guo D., Liu B. (2018). Regulatory role of rno-miR-30b-5p in IL-10 and toll-like receptor 4 expressions of T lymphocytes in experimental autoimmune uveitis in vitro. *Mediators of Inflammation*.

[96] Muhammad F., Trivett A., Wang D., Lee D. J. (2019). Tissue-specific production of microRNA-155 inhibits melanocortin 5 receptor-dependent suppressor macrophages to promote experimental autoimmune uveitis. *European Journal of Immunology*.

[97] Peng Y., Luo X., Chen Y. (2020). LncRNA and mRNA expression profile of peripheral blood mononuclear cells in primary Sjögren's syndrome patients. *Scientific Reports*.

[98] Cha S., Mona M., Lee K. E., Kim D. H., Han K. (2018). MicroRNAs in autoimmune Sjögren's syndrome. *Genomics & Informatics*.

[99] Li N., Gao Z., Zhao L. (2022). MSC-derived small extracellular vesicles attenuate autoimmune dacryoadenitis by promoting M2 macrophage polarization and inducing Tregs *via* miR-100-5p. *Frontiers in Immunology*.

[100] Fairweather D., Rose N. R. (2004). Women and autoimmune diseases. *Emerging Infectious Diseases*.

[101] Cooper G. S., Stroehla B. C. (2003). The epidemiology of autoimmune diseases. *Autoimmunity Reviews*.

[102] Ngo S. T., Steyn F. J., McCombe P. A. (2014). Gender differences in autoimmune disease. *Frontiers in Neuroendocrinology*.

[103] Moorman C. D., Sohn S. J., Phee H. (2021). Emerging therapeutics for immune tolerance: tolerogenic vaccines, T cell therapy, and IL-2 therapy. *Frontiers in Immunology*.

[104] Moulton V. R. (2018). Sex hormones in acquired immunity and autoimmune disease. *Frontiers in Immunology*.

[105] Richard-Eaglin A., Smallheer B. A. (2018). Immunosuppressive/autoimmune disorders. *The Nursing Clinics of North America*.

[106] Zucchi D., Elefante E., Calabresi E., Signorini V., Bortoluzzi A., Tani C. (2019). One year in review 2019: systemic lupus erythematosus. *Clinical and Experimental Rheumatology*.

[107] Narváez J. (2020). Systemic lupus erythematosus 2020. *Medicina Clínica*.

[108] Fava A., Petri M. (2019). Systemic lupus erythematosus: diagnosis and clinical management. *Journal of Autoimmunity*.

[109] Illescas-Montes R., Corona-Castro C. C., Melguizo-Rodríguez L., Ruiz C., Costela-Ruiz V. J. (2019). Infectious processes and systemic lupus erythematosus. *Immunology*.

[110] Fanouriakis A., Tziolos N., Bertsias G., Boumpas D. T. (2021). Update *ο*n the diagnosis and management of systemic lupus erythematosus. *Annals of the Rheumatic Diseases*.

[111] Guo G., Ye S., Xie S. (2018). The cytomegalovirus protein US31 induces inflammation through mono-macrophages in systemic lupus erythematosus by promoting NF-*κ*B2 activation. *Cell Death & Disease*.

[112] Tejon G., Valdivieso N., Flores-Santibañez F. (2022). Phenotypic and functional alterations of peritoneal macrophages in lupus-prone mice. *Molecular Biology Reports*.

[113] Labonte A. C., Kegerreis B., Geraci N. S. (2018). Identification of alterations in macrophage activation associated with disease activity in systemic lupus erythematosus. *PLoS One*.

[114] Mouton A. J., Li X., Hall M. E., Hall J. E. (2020). Obesity, hypertension, and cardiac dysfunction: novel roles of immunometabolism in macrophage activation and inflammation. *Circulation Research*.

[115] Maria N. I., Davidson A. (2020). Protecting the kidney in systemic lupus erythematosus: from diagnosis to therapy. *Nature Reviews Rheumatology*.

[116] Luan J., Fu J., Chen C. (2019). LNA-anti-miR-150 ameliorated kidney injury of lupus nephritis by inhibiting renal fibrosis and macrophage infiltration. *Arthritis Research & Therapy*.

[117] Orme J., Mohan C. (2012). Macrophage subpopulations in systemic lupus erythematosus. *Discovery Medicine*.

[118] Zhang W., Xu W., Xiong S. (2010). Blockade of Notch1 signaling alleviates murine lupus via blunting macrophage activation and M2b polarization. *Journal of Immunology*.

[119] Tanaka Y., Luo Y., O'Shea J. J., Nakayamada S. (2022). Janus kinase-targeting therapies in rheumatology: a mechanisms-based approach. *Nature Reviews Rheumatology*.

[120] Kuykendall A. T., Komrokji R. S. (2021). JAK Be Nimble: reviewing the development of JAK inhibitors and JAK inhibitor combinations for special populations of patients with myelofibrosis. *Journal of Immunotherapy and Precision Oncology*.

[121] Pang Q. M., Yang R., Zhang M. (2022). Peripheral blood-derived mesenchymal stem cells modulate macrophage plasticity through the IL-10/STAT3 pathway. *Stem Cells International*.

[122] Horuluoglu B., Bayik D., Kayraklioglu N., Goguet E., Kaplan M. J., Klinman D. M. (2019). PAM3 supports the generation of M2-like macrophages from lupus patient monocytes and improves disease outcome in murine lupus. *Journal of Autoimmunity*.

[123] Bayik D., Tross D., Haile L. A., Verthelyi D., Klinman D. M. (2017). Regulation of the maturation of human monocytes into immunosuppressive macrophages. *Blood Advances*.

[124] Zheng X., Xiao J., Jiang Q. (2022). AKT2 reduces IFN*β*1 production to modulate antiviral responses and systemic lupus erythematosus. *The EMBO Journal*.

[125] Bergtold A., Gavhane A., D'Agati V., Madaio M., Clynes R. (2006). FcR-bearing myeloid cells are responsible for triggering murine lupus nephritis. *Journal of Immunology*.

[126] Mysore V., Tahir S., Furuhashi K. (2022). Monocytes transition to macrophages within the inflamed vasculature via monocyte CCR2 and endothelial TNFR2. *The Journal of Experimental Medicine*.

[127] Anders H. J., Ryu M. (2011). Renal microenvironments and macrophage phenotypes determine progression or resolution of renal inflammation and fibrosis. *Kidney International*.

[128] Lu J., Cao Q., Zheng D. (2013). Discrete functions of M2a and M2c macrophage subsets determine their relative efficacy in treating chronic kidney disease. *Kidney International*.

[129] Liang C. L., Jiang H., Feng W. (2021). Total Glucosides of paeony ameliorate pristane-induced lupus nephritis by inducing pd-1 ligands+ macrophages via activating IL-4/STAT6/PD-L2 signaling. *Frontiers in Immunology*.

[130] Chalmers S. A., Glynn E., Garcia S. J. (2018). BTK inhibition ameliorates kidney disease in spontaneous lupus nephritis. *Clinical Immunology*.

[131] Ringheim G. E., Wampole M., Oberoi K. (2021). Bruton's tyrosine kinase (BTK) inhibitors and autoimmune diseases: making sense of BTK inhibitor specificity profiles and recent clinical trial successes and failures. *Frontiers in Immunology*.

[132] Huang Y., Xu W., Zhou R. (2021). NLRP3 inflammasome activation and cell death. *Cellular & Molecular Immunology*.

[133] Wisitpongpun P., Potup P., Usuwanthim K. (2022). Oleamide-mediated polarization of M1 macrophages and IL-1*β* production by regulating NLRP3-inflammasome activation in primary human monocyte-derived macrophages. *Frontiers in Immunology*.

[134] Zhang H., Liu L., Li L. (2018). Lentivirus-mediated knockdown of Fc*γ*RI (CD64) attenuated lupus nephritis via inhibition of NF-*κ*B regulating NLRP3 inflammasome activation in MRL/lpr mice. *Journal of Pharmacological Sciences*.

[135] Kishimoto D., Kirino Y., Tamura M. (2018). Dysregulated heme oxygenase-1low M2-like macrophages augment lupus nephritis via Bach1 induced by type I interferons. *Arthritis Research & Therapy*.

[136] Almutairi K., Nossent J., Preen D., Keen H., Inderjeeth C. (2021). The global prevalence of rheumatoid arthritis: a meta-analysis based on a systematic review. *Rheumatology International*.

[137] Almutairi K. B., Nossent J. C., Preen D. B., Keen H. I., Inderjeeth C. A. (2021). The prevalence of rheumatoid arthritis: a systematic review of population-based studies. *The Journal of Rheumatology*.

[138] Lin Y. J., Anzaghe M., Schülke S. (2020). Update on the pathomechanism, diagnosis, and treatment options for rheumatoid arthritis. *Cells*.

[139] Sparks J. A. (2019). Rheumatoid arthritis. *Annals of Internal Medicine*.

[140] Atzeni F., Talotta R., Masala I. F., Bongiovanni S., Boccassini L., Sarzi-Puttini P. (2017). Biomarkers in rheumatoid arthritis. *The Israel Medical Association Journal*.

[141] Küçükdeveci A. A. (2019). Nonpharmacological treatment in established rheumatoid arthritis. *Best Practice & Research. Clinical Rheumatology*.

[142] Chen Y., Hu Z., Cai S., Shen G., Zhong J., Dong L. (2022). Efficacy of plasma exchange on top of standard immunosuppressive therapy in adult autoimmune inflammatory rheumatic diseases-associated macrophage activation syndrome, a single center real-world analysis. *Seminars in Arthritis and Rheumatism*.

[143] Yang Y., Guo L., Wang Z. (2021). Targeted silver nanoparticles for rheumatoid arthritis therapy via macrophage apoptosis and Re-polarization. *Biomaterials*.

[144] Demarco B., Danielli S., Fischer F. A., Bezbradica J. S. (2022). How pyroptosis contributes to inflammation and fibroblast-macrophage cross-talk in rheumatoid arthritis. *Cells*.

[145] Boutet M. A., Courties G., Nerviani A. (2021). Novel insights into macrophage diversity in rheumatoid arthritis synovium. *Autoimmunity Reviews*.

[146] Kemble S., Croft A. P. (2021). Critical role of synovial tissue-resident macrophage and fibroblast subsets in the persistence of joint inflammation. *Frontiers in Immunology*.

[147] Lemke G. (2017). Phosphatidylserine is the signal for TAM receptors and their ligands. *Trends in Biochemical Sciences*.

[148] Kim H., Back J. H., Han G. (2022). Extracellular vesicle-guided in situ reprogramming of synovial macrophages for the treatment of rheumatoid arthritis. *Biomaterials*.

[149] Han C., Yang Y., Sheng Y. (2021). Glaucocalyxin B inhibits cartilage inflammatory injury in rheumatoid arthritis by regulating M1 polarization of synovial macrophages through NF-*κ*B pathway. *Aging*.

[150] Cao Y., Liu J., Huang C. (2022). Wilforlide A ameliorates the progression of rheumatoid arthritis by inhibiting M1 macrophage polarization. *Journal of Pharmacological Sciences*.

[151] Degboé Y., Rauwel B., Baron M. (2019). Polarization of rheumatoid macrophages by TNF targeting through an IL-10/STAT3 mechanism. *Frontiers in Immunology*.

[152] Zhong Y. M., Zhang L. L., Lu W. T., Shang Y. N., Zhou H. Y. (2022). Moxibustion regulates the polarization of macrophages through the IL-4/STAT6 pathway in rheumatoid arthritis. *Cytokine*.

[153] Woo S. J., Noh H. S., Lee N. Y. (2018). Myeloid sirtuin 6 deficiency accelerates experimental rheumatoid arthritis by enhancing macrophage activation and infiltration into synovium. *eBioMedicine*.

[154] Lee Y., Ka S. O., Cha H. N. (2017). Myeloid sirtuin 6 deficiency causes insulin resistance in high-fat diet-fed mice by eliciting macrophage polarization toward an M1 phenotype. *Diabetes*.

[155] Li H., Feng Y., Zheng X. (2022). M2-type exosomes nanoparticles for rheumatoid arthritis therapy via macrophage re-polarization. *Journal of Controlled Release*.

[156] Feige J., Moser T., Bieler L., Schwenker K., Hauer L., Sellner J. (2020). Vitamin D supplementation in multiple sclerosis: a critical analysis of potentials and threats. *Nutrients*.

[157] Houen G., Trier N. H., Frederiksen J. L. (2020). Epstein-Barr virus and multiple sclerosis. *Frontiers in Immunology*.

[158] Alfredsson L., Olsson T. (2019). Lifestyle and environmental factors in multiple sclerosis. *Cold Spring Harbor Perspectives in Medicine*.

[159] Tafti D., Ehsan M., Xixis K. L. (2022). Multiple Sclerosis. 2022 Apr 9. *StatPearls*.

[160] Hauser S. L., Cree B. A. C. (2020). Treatment of multiple sclerosis: a review. *The American Journal of Medicine*.

[161] Kamińska J., Koper O. M., Piechal K., Kemona H. (2017). Multiple sclerosis - etiology and diagnostic potential. *Postȩpy Higieny i Medycyny Doświadczalnej*.

[162] Vogel D. Y., Vereyken E. J., Glim J. E. (2013). Macrophages in inflammatory multiple sclerosis lesions have an intermediate activation status. *Journal of Neuroinflammation*.

[163] Leuti A., Talamonti E., Gentile A. (2021). Macrophage plasticity and polarization are altered in the experimental model of multiple sclerosis. *Biomolecules*.

[164] Jiang F., Liu X., Cui X. (2022). Circ_0000518 promotes macrophage/microglia M1 polarization via the FUS/CaMKK*β*/AMPK pathway to aggravate multiple sclerosis. *Neuroscience*.

[165] Kamma E., Lasisi W., Libner C., Ng H. S., Plemel J. R. (2022). Central nervous system macrophages in progressive multiple sclerosis: relationship to neurodegeneration and therapeutics. *Journal of Neuroinflammation*.

[166] Kuntzel T., Bagnard D. (2022). Manipulating macrophage/microglia polarization to treat glioblastoma or multiple sclerosis. *Pharmaceutics*.

[167] Li B., Tan T. B., Wang L., Zhao X. Y., Tan G. J. (2019). p38MAPK/SGK1 signaling regulates macrophage polarization in experimental autoimmune encephalomyelitis. *Aging*.

[168] Shi M., Mi L., Li F. (2022). Fluvoxamine confers neuroprotection via inhibiting infiltration of peripheral leukocytes and M1 polarization of microglia/macrophages in a mouse model of traumatic brain injury. *Journal of Neurotrauma*.

[169] Wu Y., Hu Y., Wang B. (2020). Dopamine uses the DRD5-ARRB2-PP2A signaling axis to block the TRAF6-mediated NF-*κ*B pathway and suppress systemic inflammation. *Molecular Cell*.

[170] Yoshioka Y., Sugino Y., Shibagaki F., Yamamuro A., Ishimaru Y., Maeda S. (2020). Dopamine attenuates lipopolysaccharide-induced expression of proinflammatory cytokines by inhibiting the nuclear translocation of NF-*κ*B p65 through the formation of dopamine quinone in microglia. *European Journal of Pharmacology*.

[171] Liu X., Zhang X., Niu X. (2022). Mdivi-1 modulates macrophage/microglial polarization in mice with EAE via the inhibition of the TLR2/4-GSK3*β*-NF-*κ*B inflammatory signaling axis. *Molecular Neurobiology*.

[172] Liu S., Dong C., Ubogu E. E. (2018). Immunotherapy of Guillain-Barré syndrome. *Human Vaccines & Immunotherapeutics*.

[173] Leonhard S. E., Mandarakas M. R., Gondim F. A. A. (2019). Diagnosis and management of Guillain-Barré syndrome in ten steps. *Nature Reviews Neurology*.

[174] Caspi R. R., Silver P. B., Luger D. (2008). Mouse models of experimental autoimmune uveitis. *Ophthalmic Research*.

[175] Nyati K. K., Prasad K. N., Rizwan A., Verma A., Paliwal V. K. (2011). TH1 and TH2 response to Campylobacter jejuni antigen in Guillain-Barre syndrome. *Archives of Neurology*.

[176] Shen D., Chu F., Lang Y. (2018). Beneficial or harmful role of macrophages in Guillain-Barré syndrome and experimental autoimmune neuritis. *Mediators of Inflammation*.

[177] Du T., Yang C. L., Ge M. R. (2020). M1 macrophage derived exosomes aggravate experimental autoimmune neuritis via modulating Th1 response. *Frontiers in Immunology*.

[178] Xu J., Chi F., Tsukamoto H. (2015). Notch signaling and M1 macrophage activation in obesity-alcohol synergism. *Clinics and Research in Hepatology and Gastroenterology*.

[179] Xu L., Li L., Zhang C. Y., Schluesener H., Zhang Z. Y. (2019). Natural diterpenoid oridonin ameliorates experimental autoimmune neuritis by promoting anti-inflammatory macrophages through blocking Notch pathway. *Frontiers in Neuroscience*.

[180] Shen D., Chu F., Lang Y. (2021). Nuclear factor kappa B inhibitor suppresses experimental autoimmune neuritis in mice via declining macrophages polarization to M1 type. *Clinical and Experimental Immunology*.

[181] Fu X., Chen Y., Chen D. (2021). The role of gut microbiome in autoimmune uveitis. *Ophthalmic Research*.

[182] Li H., Zhu L., Wang R. (2021). Therapeutic effect of IL-38 on experimental autoimmune uveitis: reprogrammed immune cell landscape and reduced Th17 cell pathogenicity. *Investigative Ophthalmology & Visual Science*.

[183] Mérida S., Palacios E., Navea A., Bosch-Morell F. (2015). Macrophages and uveitis in experimental animal models. *Mediators of Inflammation*.

[184] Wang C., Zhou W., Su G., Hu J., Yang P. (2022). Progranulin suppressed autoimmune uveitis and autoimmune neuroinflammation by inhibiting Th1/Th17 cells and promoting Treg cells and M2 macrophages. *Neurology - Neuroimmunology Neuroinflammation*.

[185] Yin X., Wei H., Wu S. (2020). DAPT reverses the Th17/Treg imbalance in experimental autoimmune uveitis in vitro via inhibiting Notch signaling pathway. *International Immunopharmacology*.

[186] Yin X., Qiu Y., Li Z. (2021). Longdan Xiegan decoction alleviates experimental autoimmune uveitis in rats by inhibiting Notch signaling pathway activation and Th17 cell differentiation. *Biomedicine & Pharmacotherapy*.

[187] Yin X., Guo L., Zhou M., Qiu Y., Bi H., Guo D. (2021). Regulatory role of Longdan Xiegan decoction in polarization balance of M1/M2 macrophages in rats with experimental autoimmune uveitis. *Recent Advances in Ophthalmology*.

[188] Sun S. C. (2011). Non-canonical NF-*κ*B signaling pathway. *Cell Research*.

[189] Liu Y., Zhao C., Meng J. (2022). Galectin-3 regulates microglial activation and promotes inflammation through TLR4/MyD88/NF-kB in experimental autoimmune uveitis. *Clinical Immunology*.

[190] Liu Y., Kitaichi N., Wu D. (2020). Attenuation of experimental autoimmune uveoretinitis in mice by IKK*β* inhibitor IMD-0354. *Biochemical and Biophysical Research Communications*.

[191] Chen Y., Li Z., Li H. (2020). Apremilast regulates the Teff/Treg balance to ameliorate uveitis via PI3K/AKT/FoxO1 signaling pathway. *Frontiers in Immunology*.

[192] Wang G., Li X., Li N. (2022). Icariin alleviates uveitis by targeting peroxiredoxin 3 to modulate retinal microglia M1/M2 phenotypic polarization. *Redox Biology*.

[193] Mavragani C. P., Moutsopoulos H. M. (2014). Sjögren syndrome. *CMAJ*.

[194] Ogawa Y., Shimizu E., Tsubota K. (2018). Interferons and dry eye in Sjögren's syndrome. *International Journal of Molecular Sciences*.

[195] Båve U., Nordmark G., Lövgren T. (2005). Activation of the type I interferon system in primary Sjögren's syndrome: a possible etiopathogenic mechanism. *Arthritis and Rheumatism*.

[196] Emamian E. S., Leon J. M., Lessard C. J. (2009). Peripheral blood gene expression profiling in Sjögren's syndrome. *Genes and Immunity*.

[197] Chivasso C., Sarrand J., Perret J., Delporte C., Soyfoo M. S. (2021). The involvement of innate and adaptive immunity in the initiation and perpetuation of Sjögren's syndrome. *International Journal of Molecular Sciences*.

[198] Baturone R., Soto M. J., Márquez M. (2009). Health-related quality of life in patients with primary Sjögren's syndrome: relationship with serum levels of proinflammatory cytokines. *Scandinavian Journal of Rheumatology*.

[199] Lu X., Li N., Zhao L. (2020). Human umbilical cord mesenchymal stem cells alleviate ongoing autoimmune dacryoadenitis in rabbits via polarizing macrophages into an anti-inflammatory phenotype. *Experimental Eye Research*.

[200] Raes G., Brys L., Dahal B. K. (2005). Macrophage galactose-type C-type lectins as novel markers for alternatively activated macrophages elicited by parasitic infections and allergic airway inflammation. *Journal of Leukocyte Biology*.

[201] Frangogiannis N. (2020). Transforming growth factor-*β* in tissue fibrosis. *The Journal of Experimental Medicine*.

[202] Hata A., Chen Y. G. (2016). TGF-*β* signaling from receptors to Smads. *Cold Spring Harbor Perspectives in Biology*.

[203] Su J., Morgani S. M., David C. J. (2020). TGF-*β* orchestrates fibrogenic and developmental EMTs via the RAS effector RREB1. *Nature*.

[204] Kim J., Kim Y. S., Park S. H. (2021). Metformin as a treatment strategy for Sjögren's syndrome. *International Journal of Molecular Sciences*.

[205] Long D., Chen Y., Wu H., Zhao M., Lu Q. (2019). Clinical significance and immunobiology of IL-21 in autoimmunity. *Journal of Autoimmunity*.

[206] Ren Y., Cui G., Gao Y. (2021). Research progress on inflammatory mechanism of primary Sjögren syndrome. *Zhejiang Da Xue Xue Bao. Yi Xue Ban*.

[207] Kiripolsky J., Romano R. A., Kasperek E. M., Yu G., Kramer J. M. (2020). Activation of Myd88-dependent TLRs mediates local and systemic inflammation in a mouse model of primary Sjögren's syndrome. *Frontiers in Immunology*.

[208] Cutolo M., Soldano S., Smith V. (2019). Pathophysiology of systemic sclerosis: current understanding and new insights. *Expert Review of Clinical Immunology*.

[209] Brown M., O'Reilly S. (2019). The immunopathogenesis of fibrosis in systemic sclerosis. *Clinical and Experimental Immunology*.

[210] Liu C., Tang J., Liu S. (2022). Cathepsin B/NLRP3/GSDMD axis-mediated macrophage pyroptosis induces inflammation and fibrosis in systemic sclerosis. *Journal of Dermatological Science*.

[211] Lescoat A., Lelong M., Jeljeli M. (2020). Combined anti-fibrotic and anti-inflammatory properties of JAK-inhibitors on macrophages in vitro and in vivo: perspectives for scleroderma-associated interstitial lung disease. *Biochemical Pharmacology*.

[212] Rudnik M., Hukara A., Kocherova I. (2021). Elevated fibronectin levels in profibrotic CD14+ monocytes and CD14+ macrophages in systemic sclerosis. *Frontiers in Immunology*.

[213] Huang M., Smith A., Watson M. (2022). Self-assembled human skin equivalents model macrophage activation of cutaneous fibrogenesis in systemic sclerosis. *Arthritis & Rhematology*.

[214] Sun C., Cai D., Chen S. Y. (2022). ADAR1 promotes systemic sclerosis *via* modulating classic macrophage activation. *Frontiers in Immunology*.

[215] Wang Y., Zhang L., Wu G. R. (2021). MBD2 serves as a viable target against pulmonary fibrosis by inhibiting macrophage M2 program. *Science Advances*.

[216] Abron J. D., Singh N. P., Price R. L., Nagarkatti M., Nagarkatti P. S., Singh U. P. (2018). Genistein induces macrophage polarization and systemic cytokine to ameliorate experimental colitis. *PLoS One*.

[217] Li Y., Huang B., Jiang X. (2018). Mucosal-associated invariant T cells improve nonalcoholic fatty liver disease through regulating macrophage polarization. *Frontiers in Immunology*.

[218] Nguyen V. T., Farman N., Palacios-Ramirez R. (2020). Cutaneous wound healing in diabetic mice is improved by topical mineralocorticoid receptor blockade. *The Journal of Investigative Dermatology*.

